# Supplementary material for: Deciphering lung adenocarcinoma evolution and the role of LINE-1 retrotransposition
Source: bioRxiv. 2025 Mar 16:2025.03.14.643063. Preprint. [Version 1] doi: 10.1101/2025.03.14.643063 (PMC11952568; doi:10.1101/2025.03.14.643063)

Supplementary Fig. 1

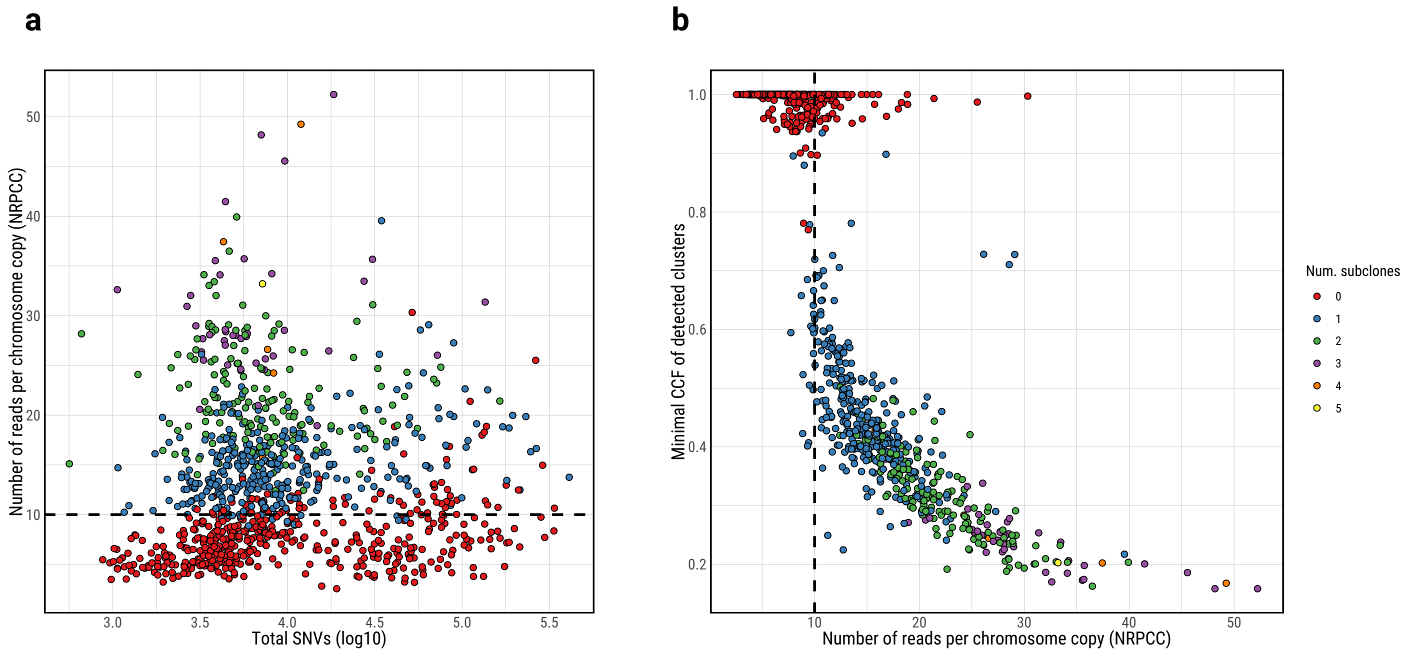

Supplementary Fig. 2

a

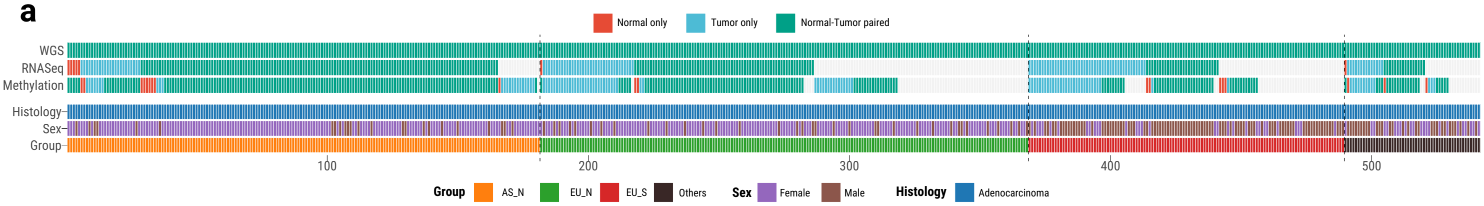

b

Group	n	percent
AS_N	180	33.2%
EU_N	184	34.0%
EU_S	120	22.1%
Others	58	10.7%

c

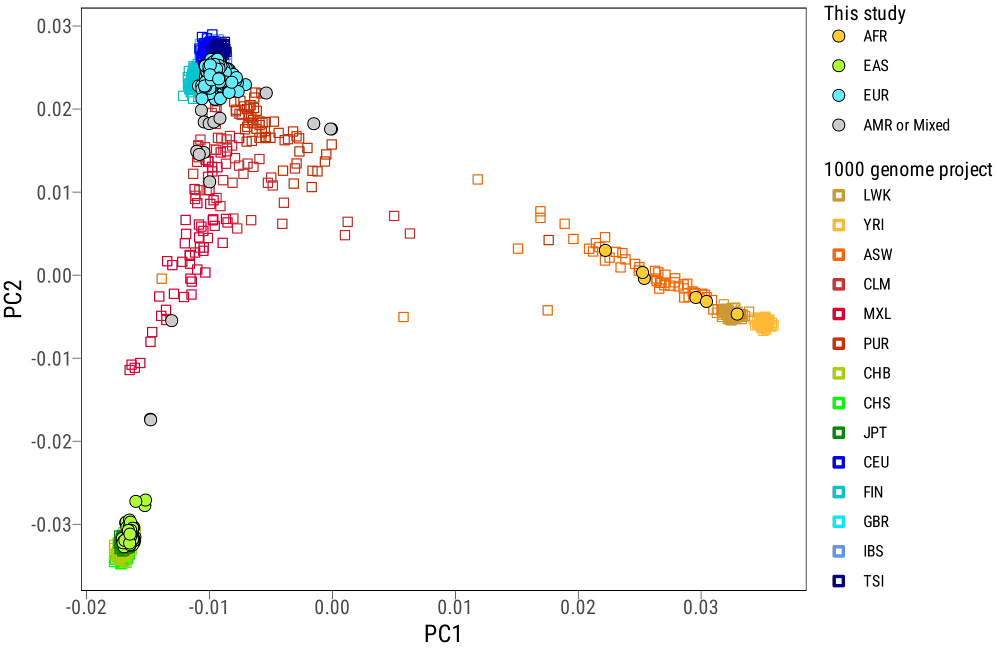

Supplementary Fig. 3

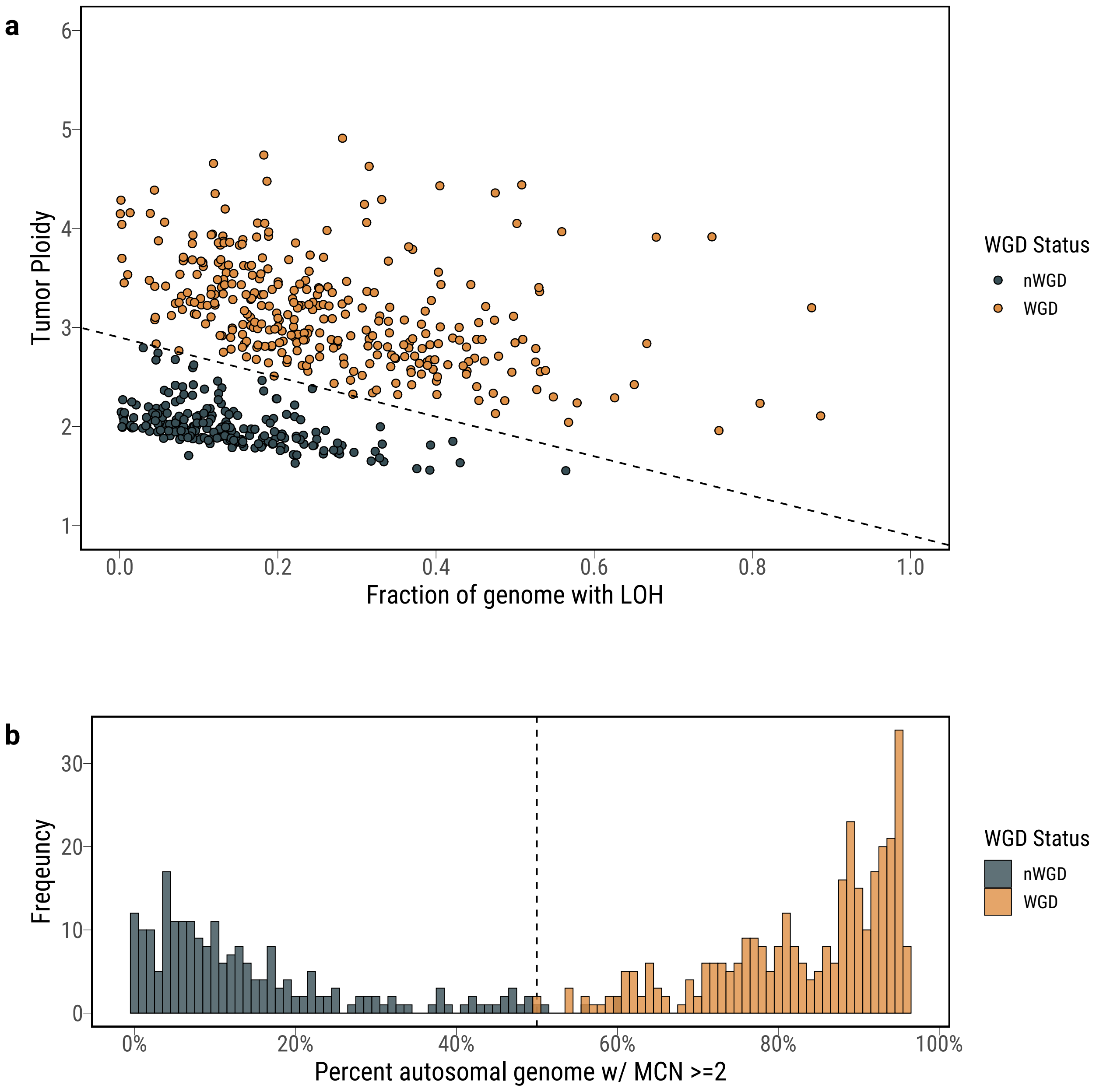

Supplementary Fig. 4

a

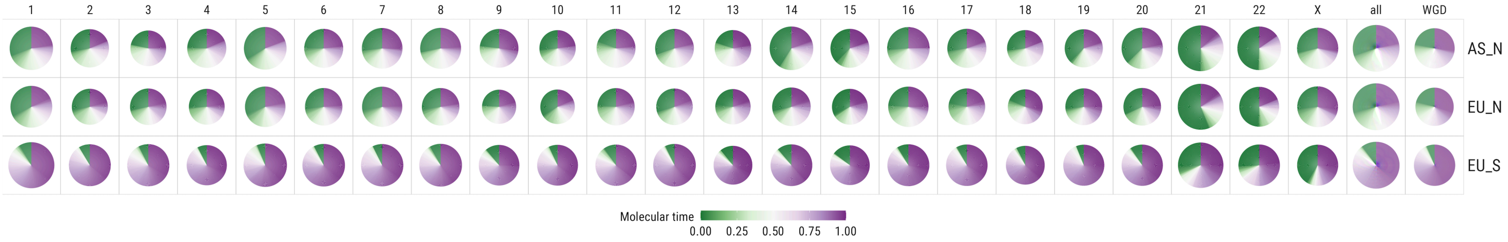

b

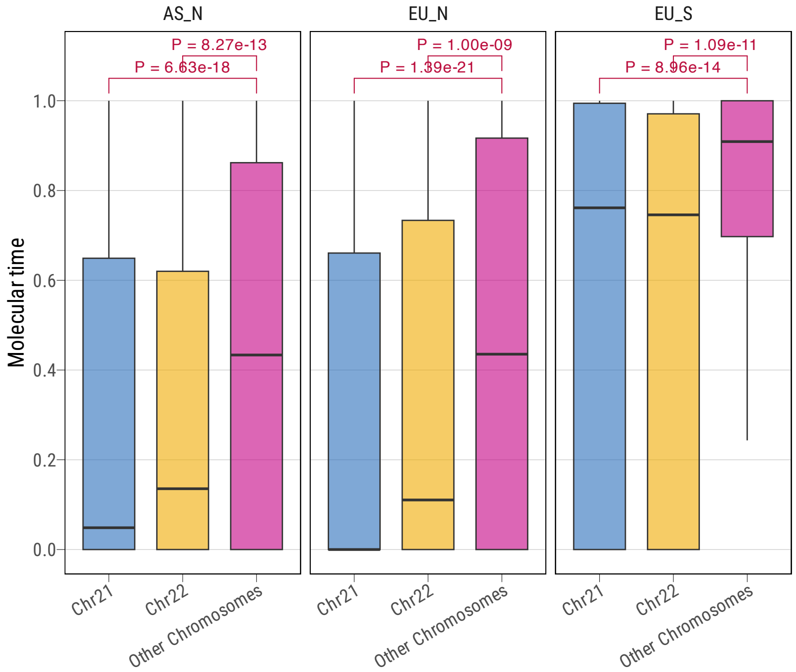

c

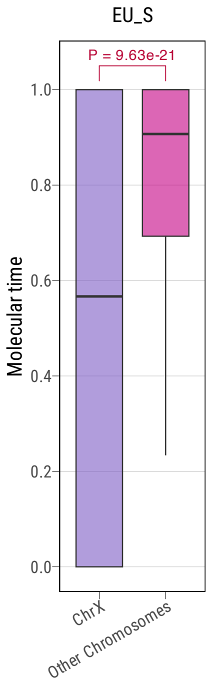

Supplementary Fig. 5

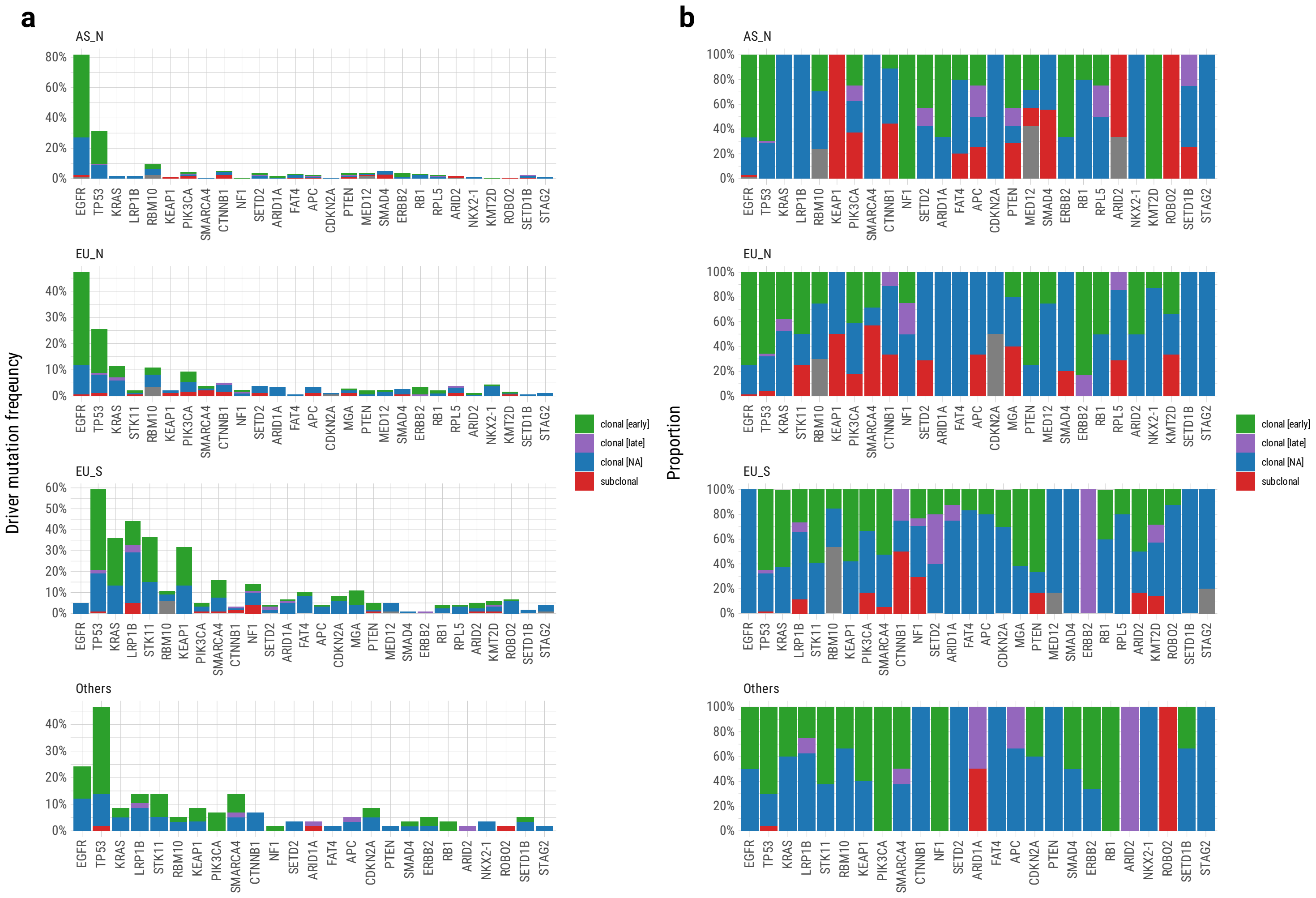

Supplementary Fig. 6

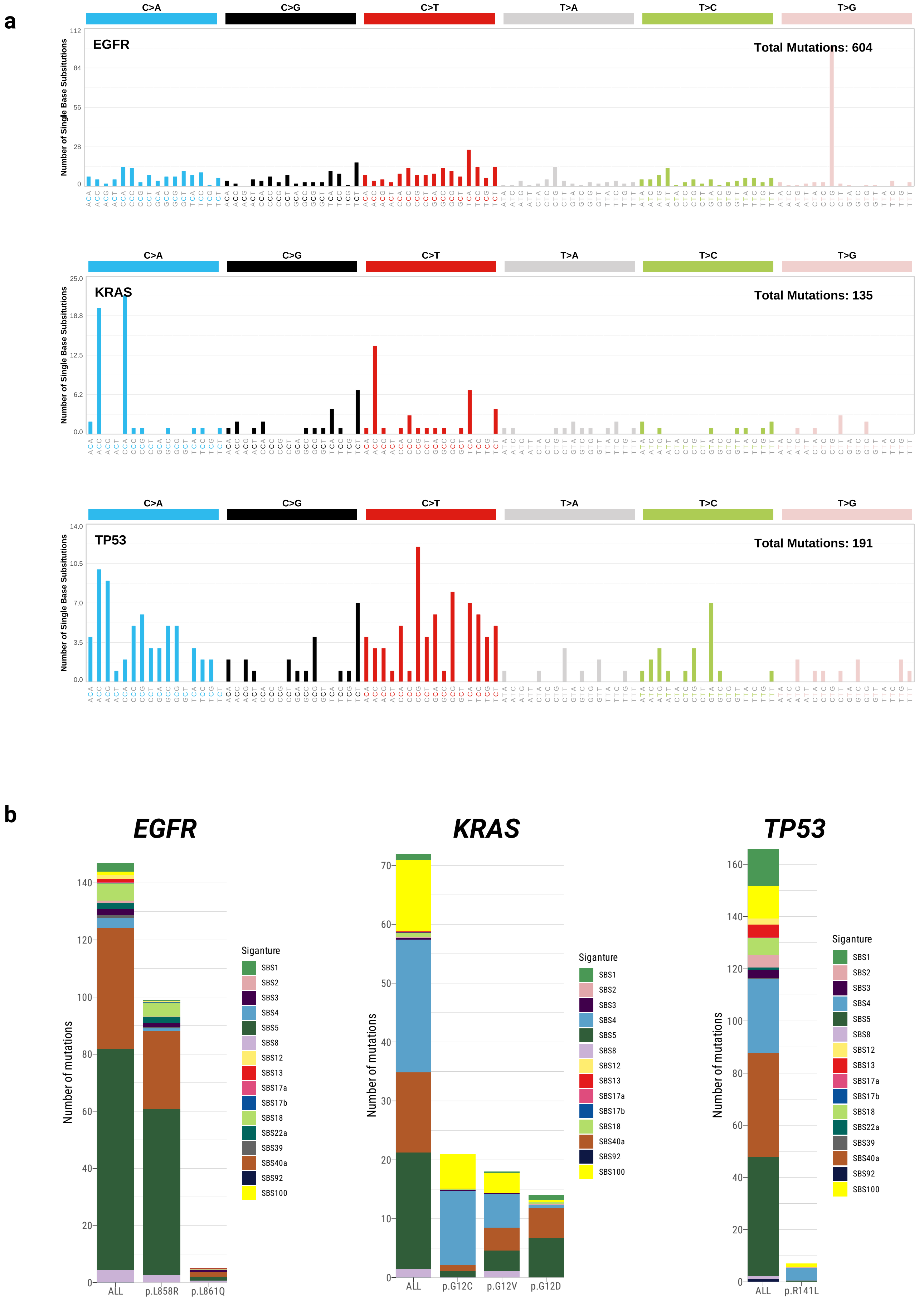

Supplementary Fig. 7

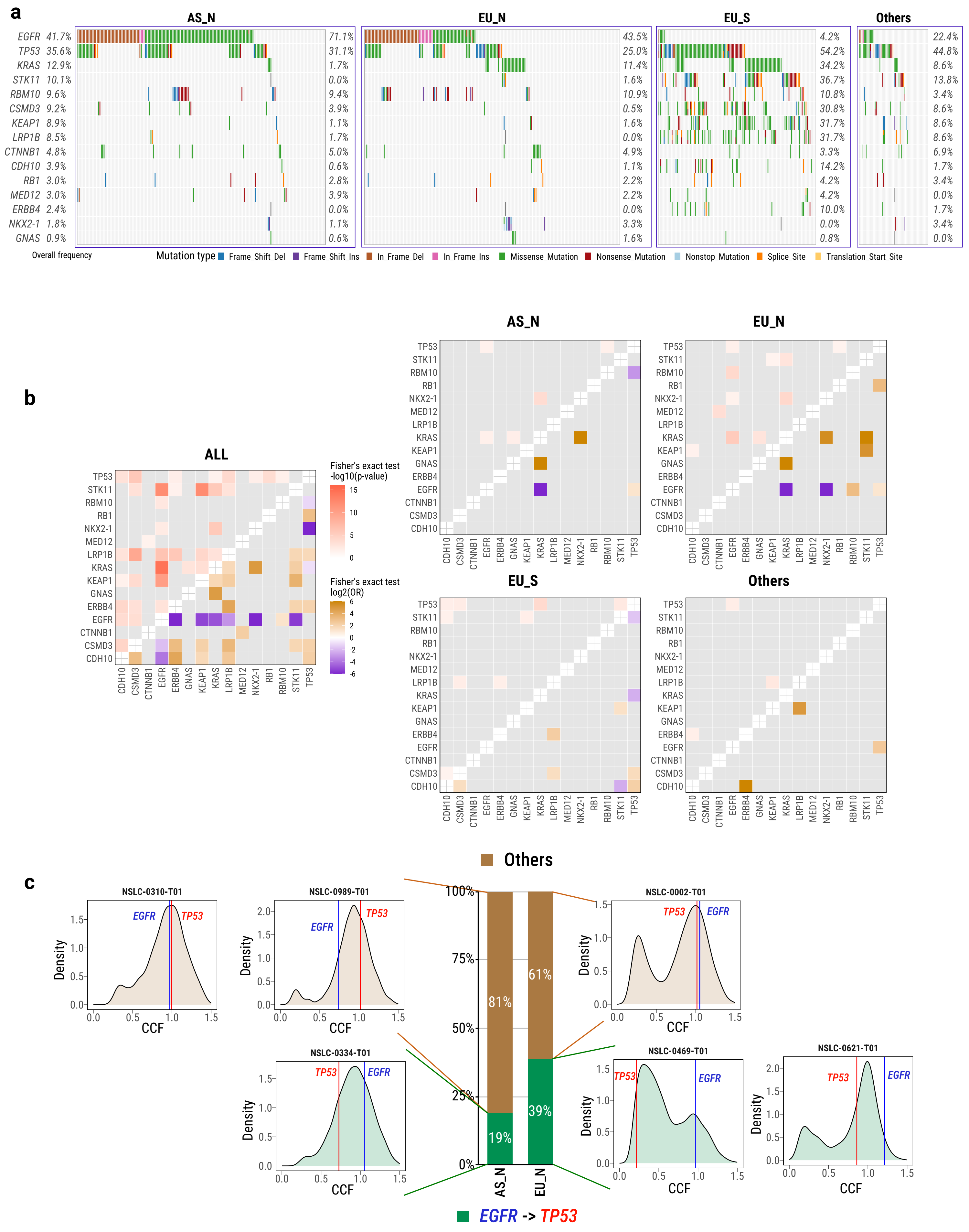

Supplementary Fig. 8

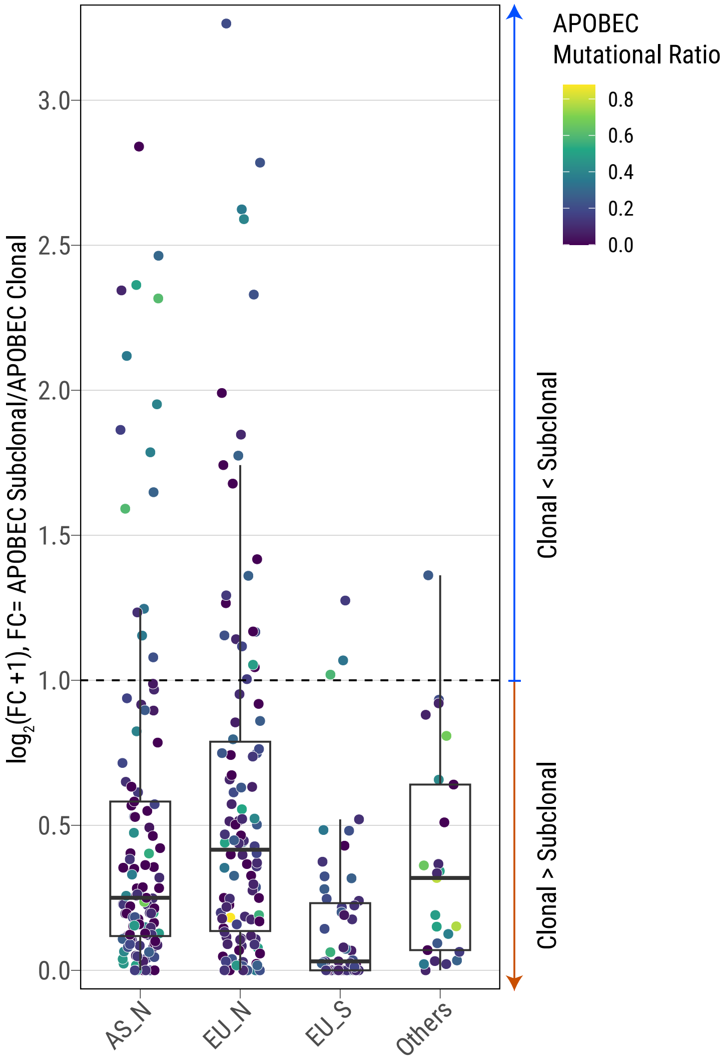

Supplementary Fig. 9

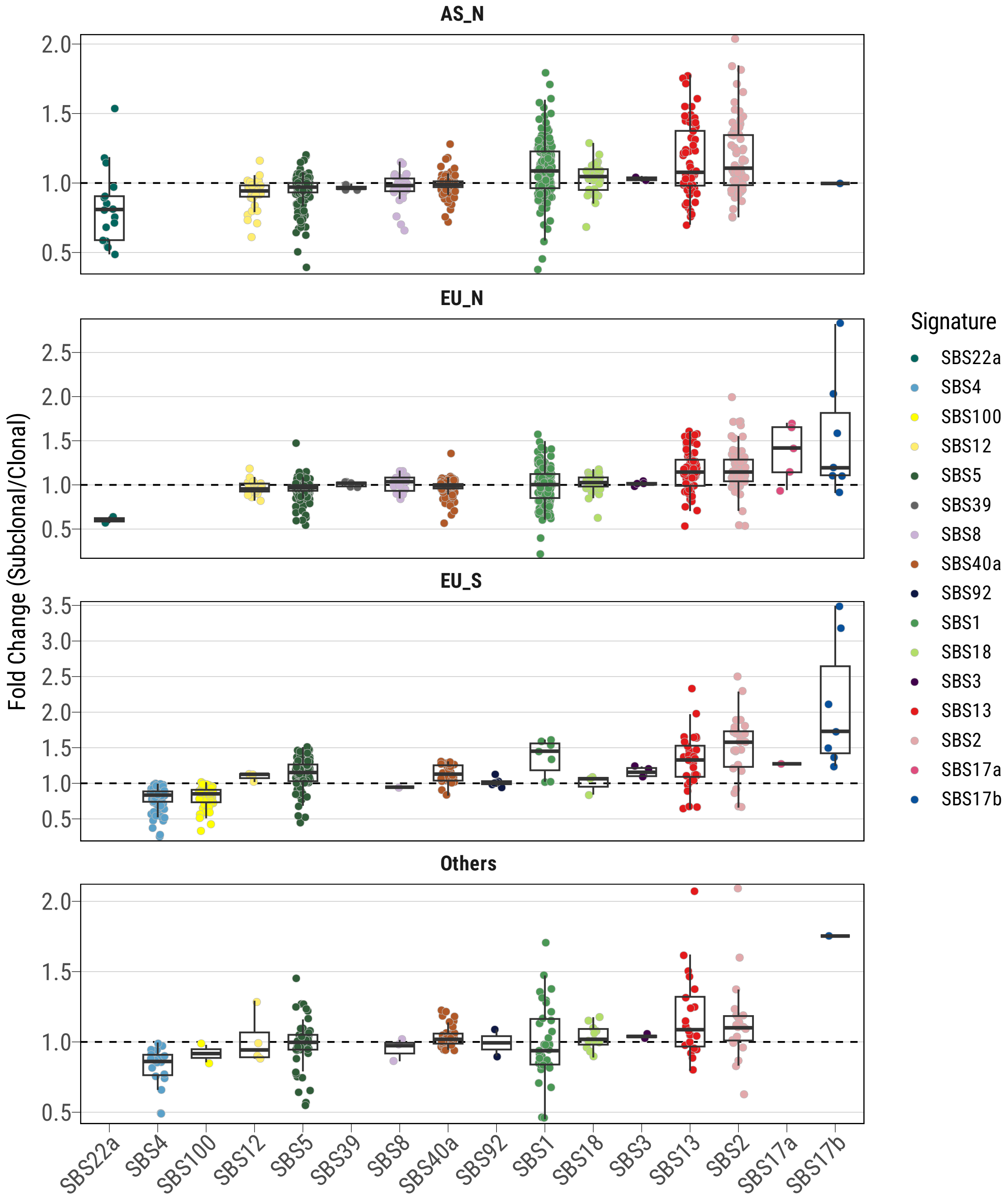

Supplementary Fig. 10

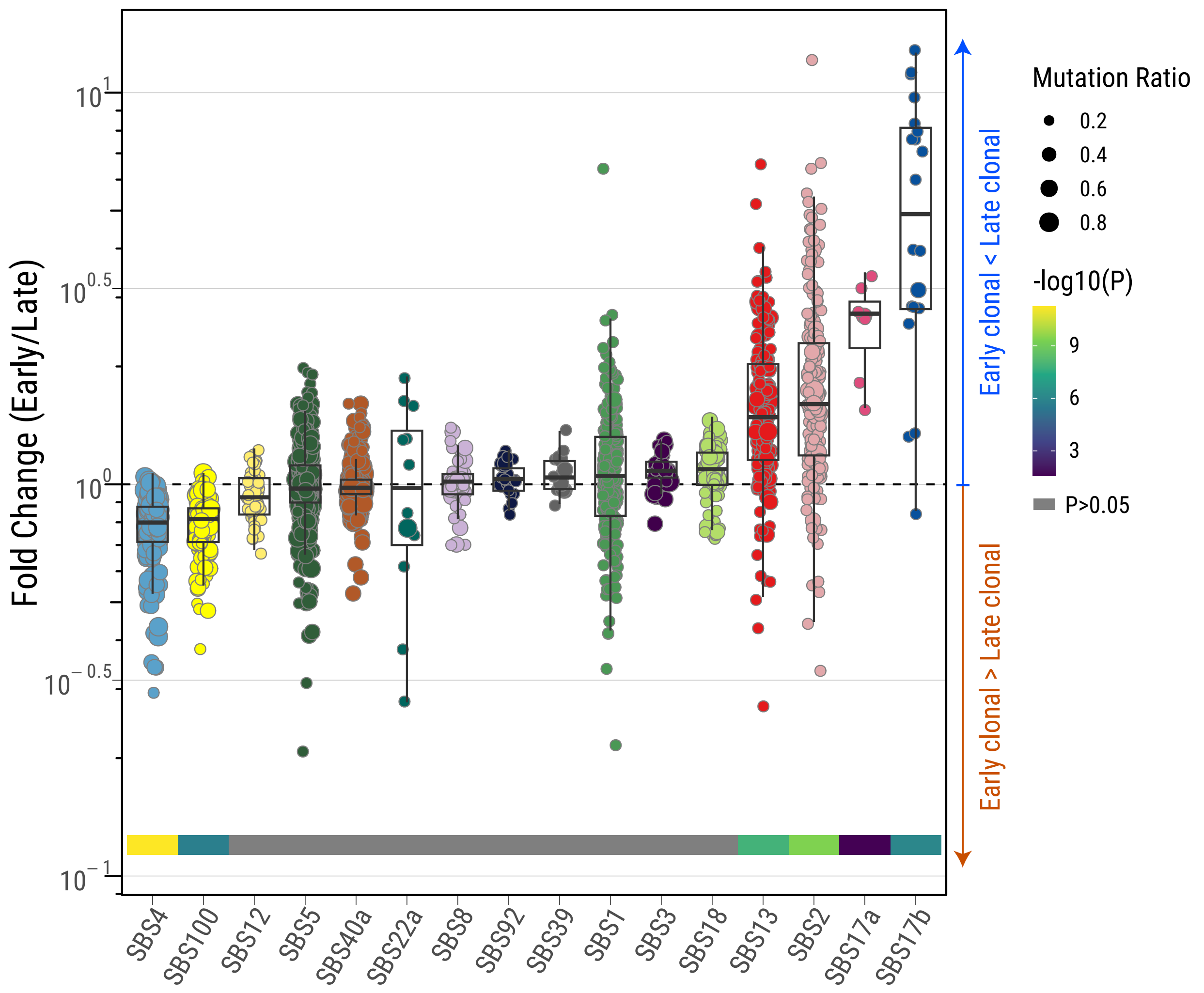

Supplementary Fig. 11

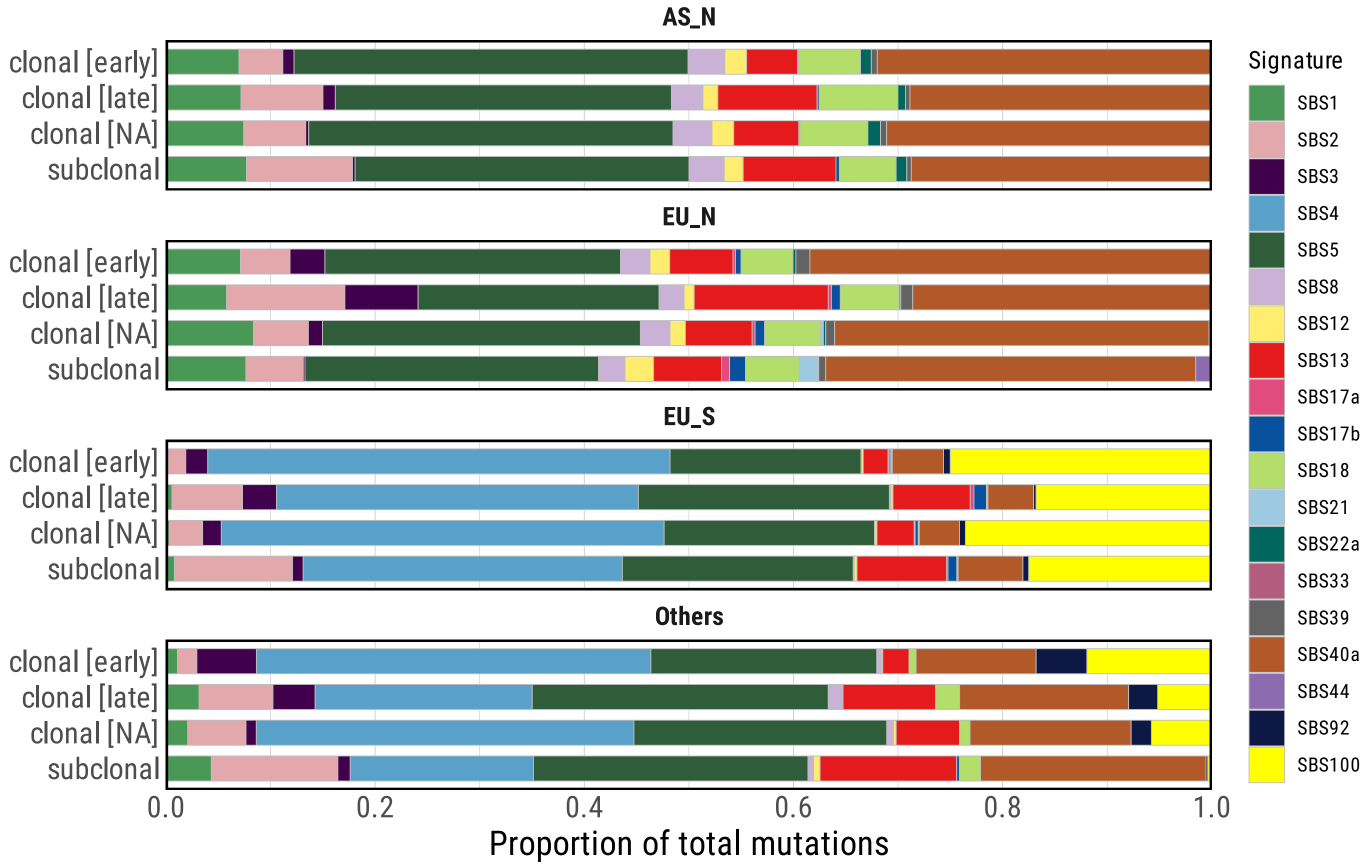

Supplementary Fig. 12

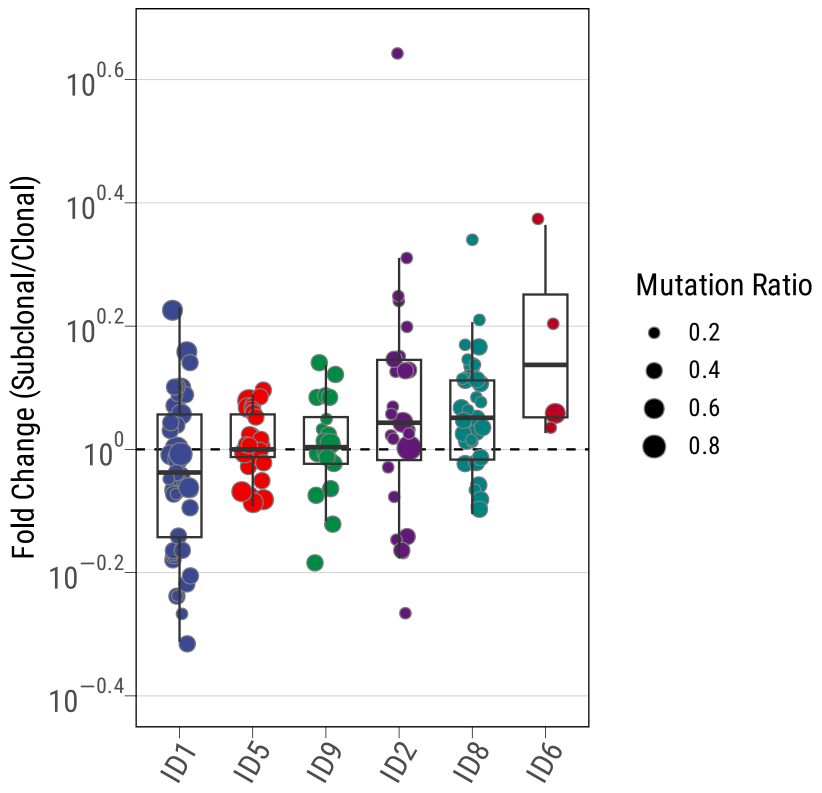

Supplementary Fig. 13

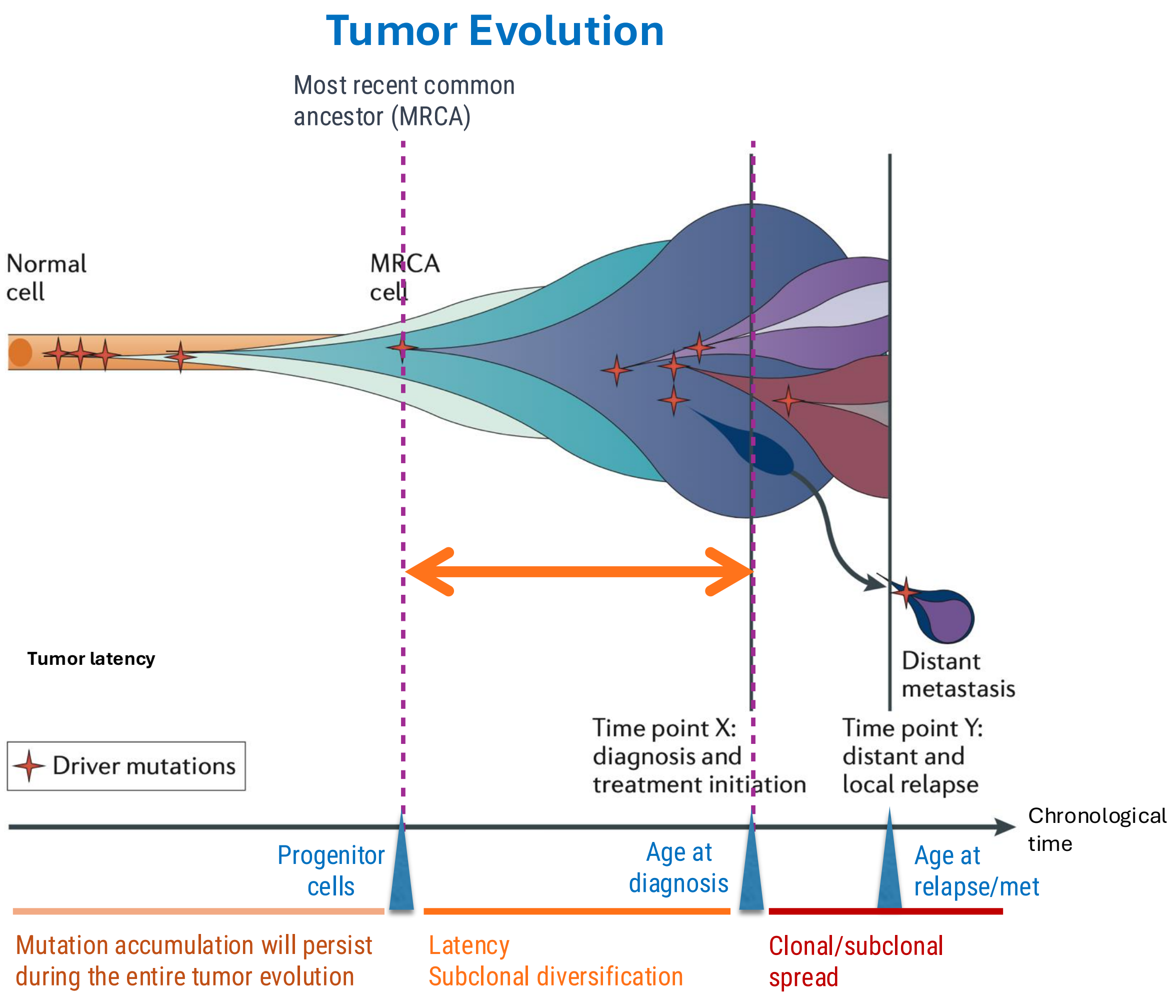

Figure adapted from Yates and Campbell, *Nature Reviews Genetics*, 2012

Supplementary Fig. 14

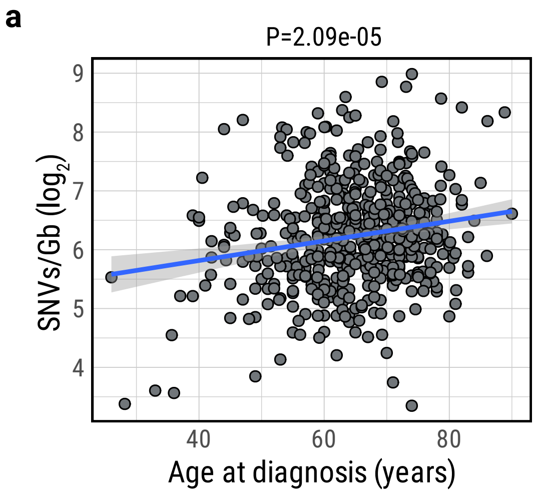

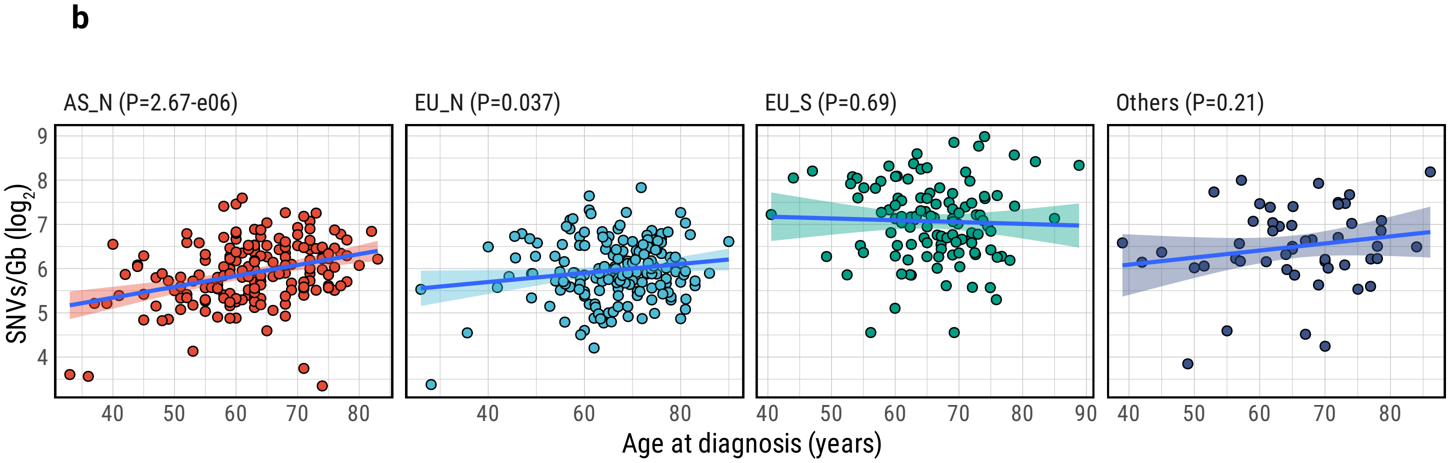

Supplementary Fig. 15

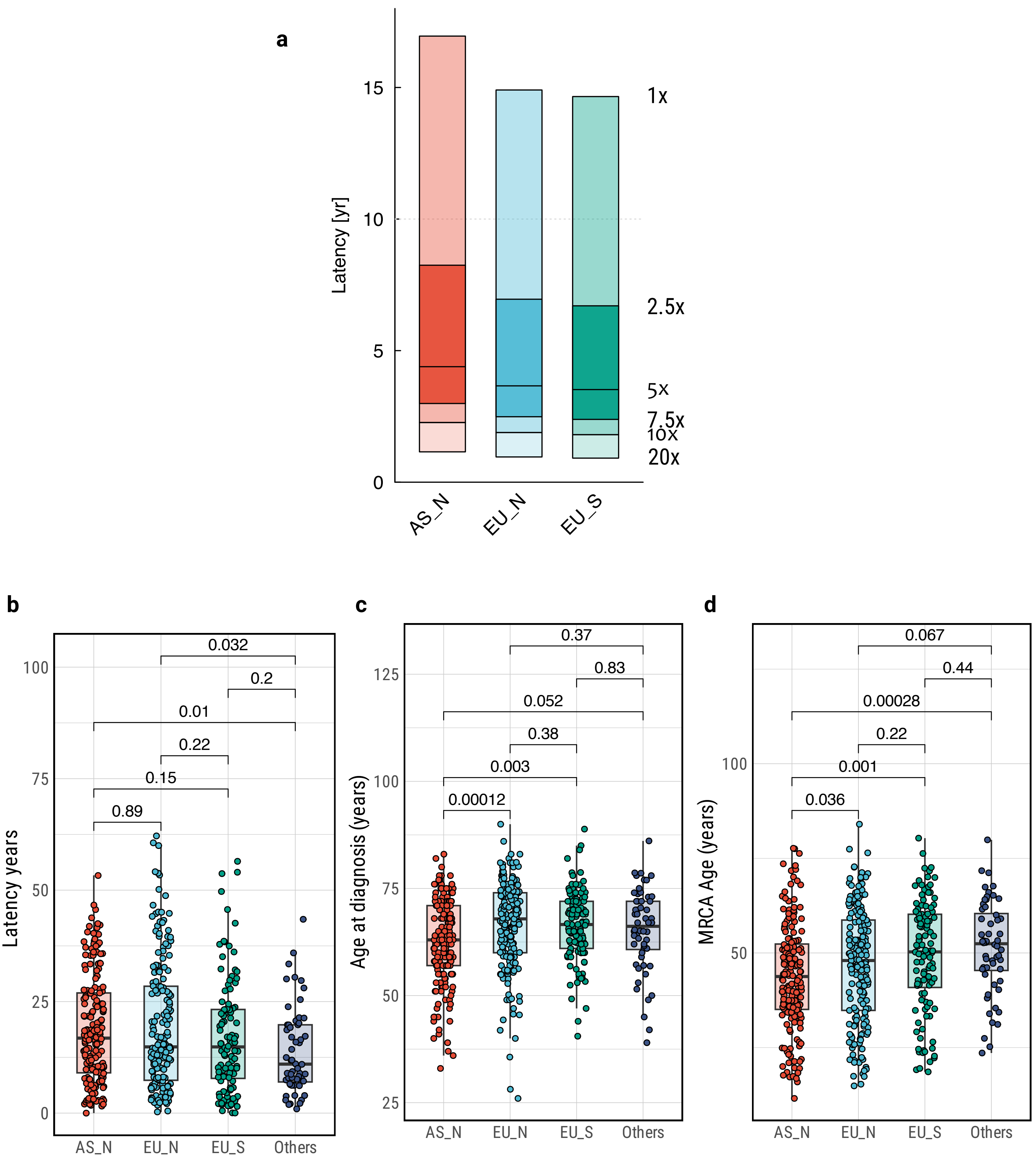

Supplementary Fig. 16

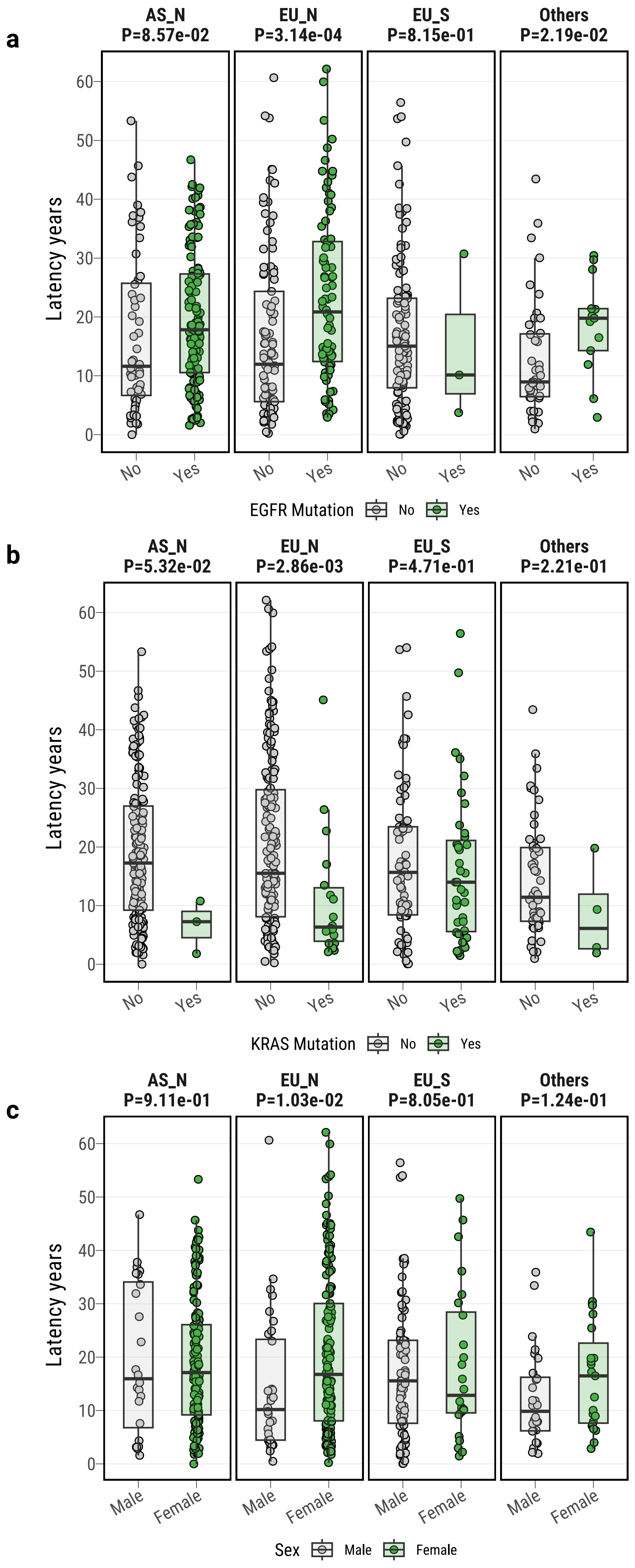

Supplementary Fig. 17

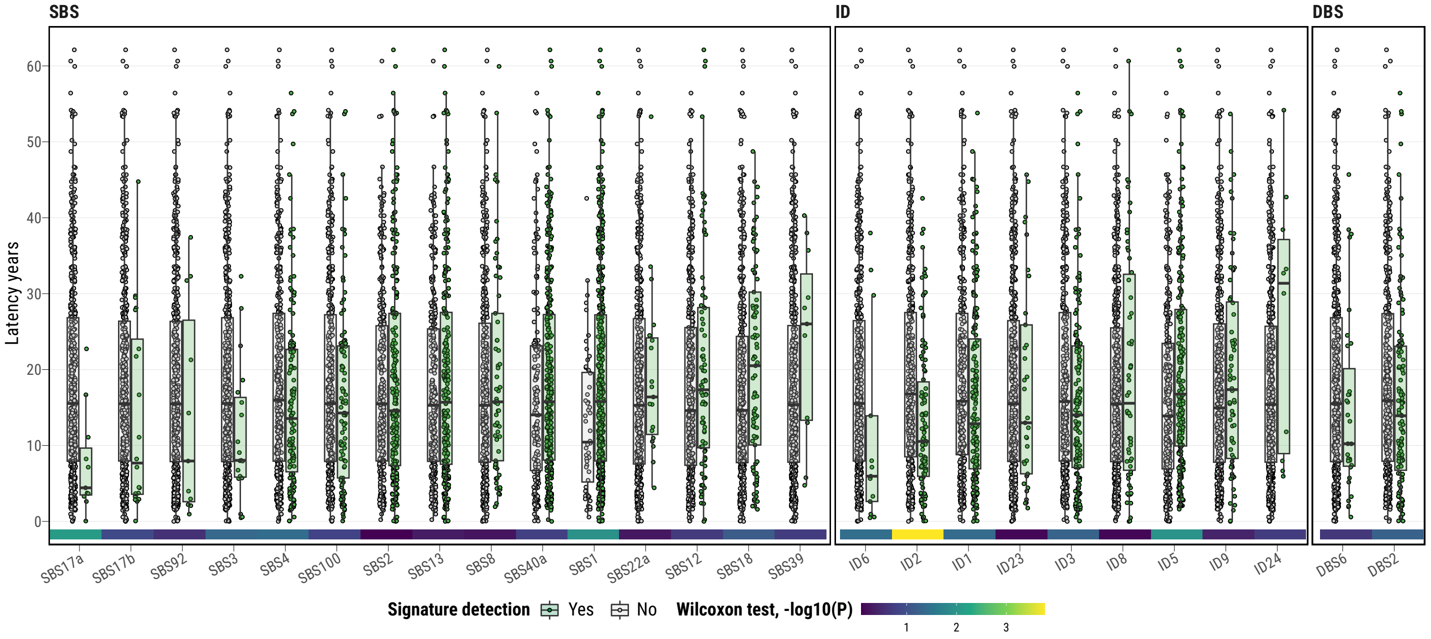

Supplementary Fig. 18

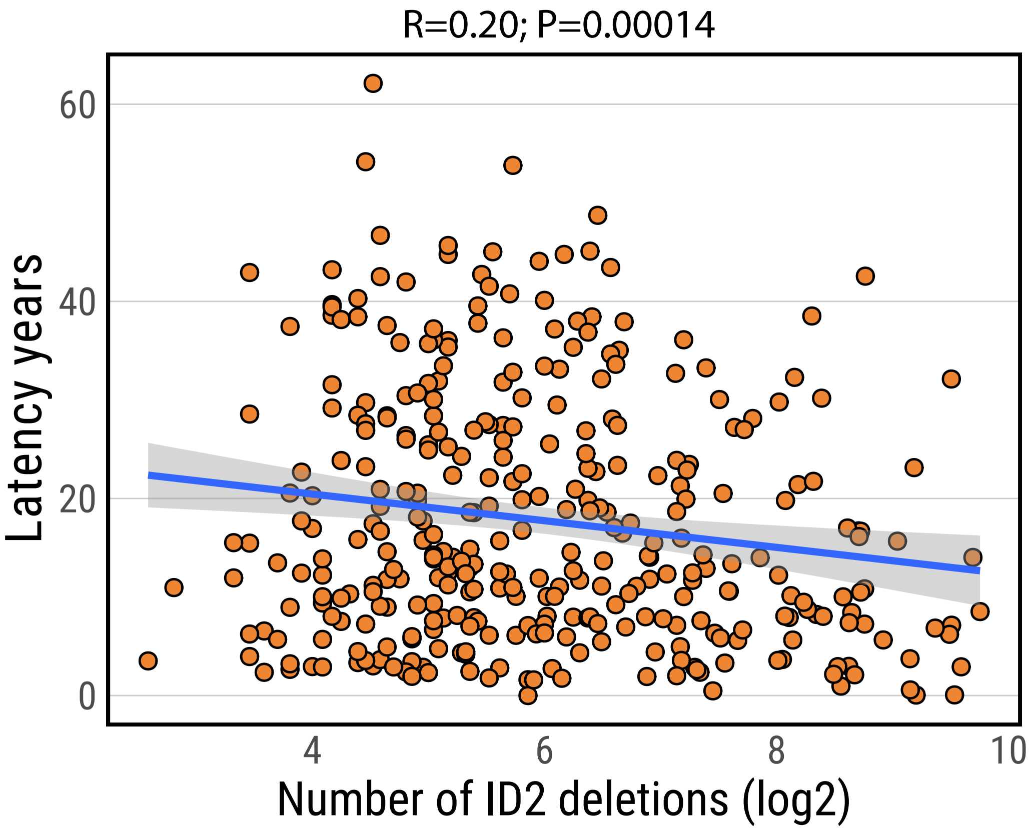

Supplementary Fig. 19

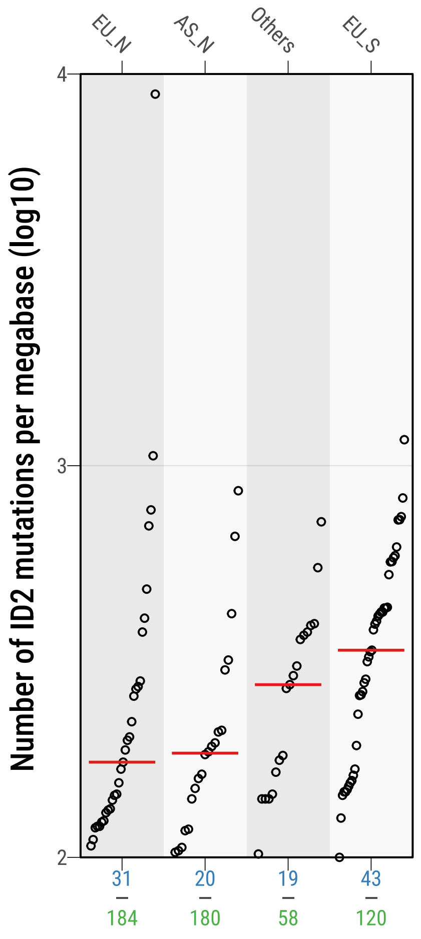

Supplementary Fig. 20

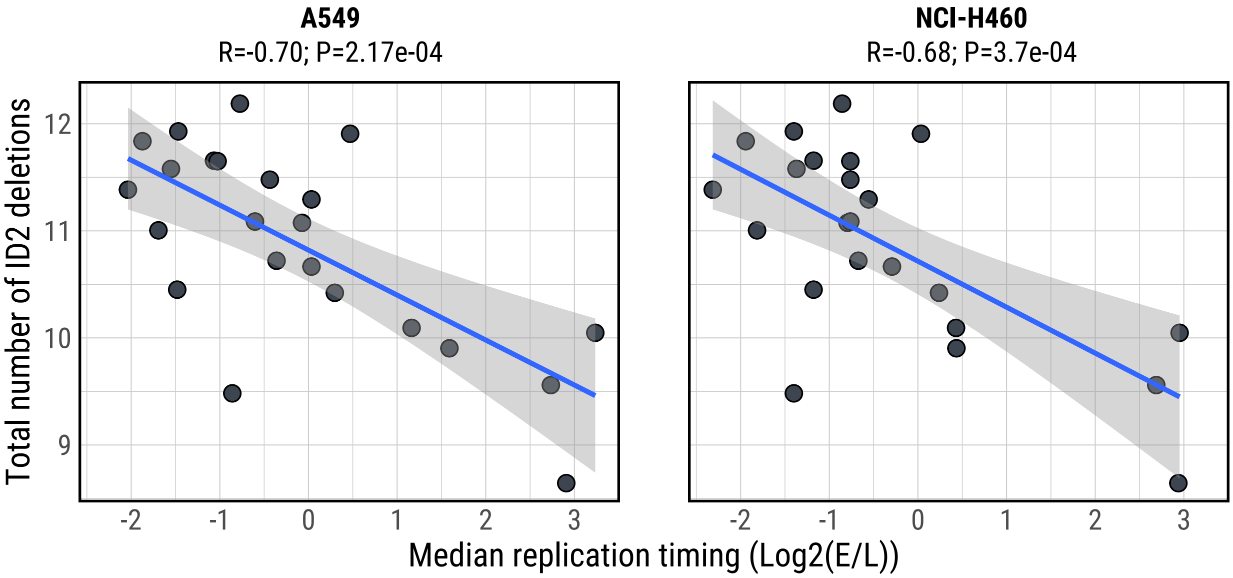

Supplementary Fig. 21

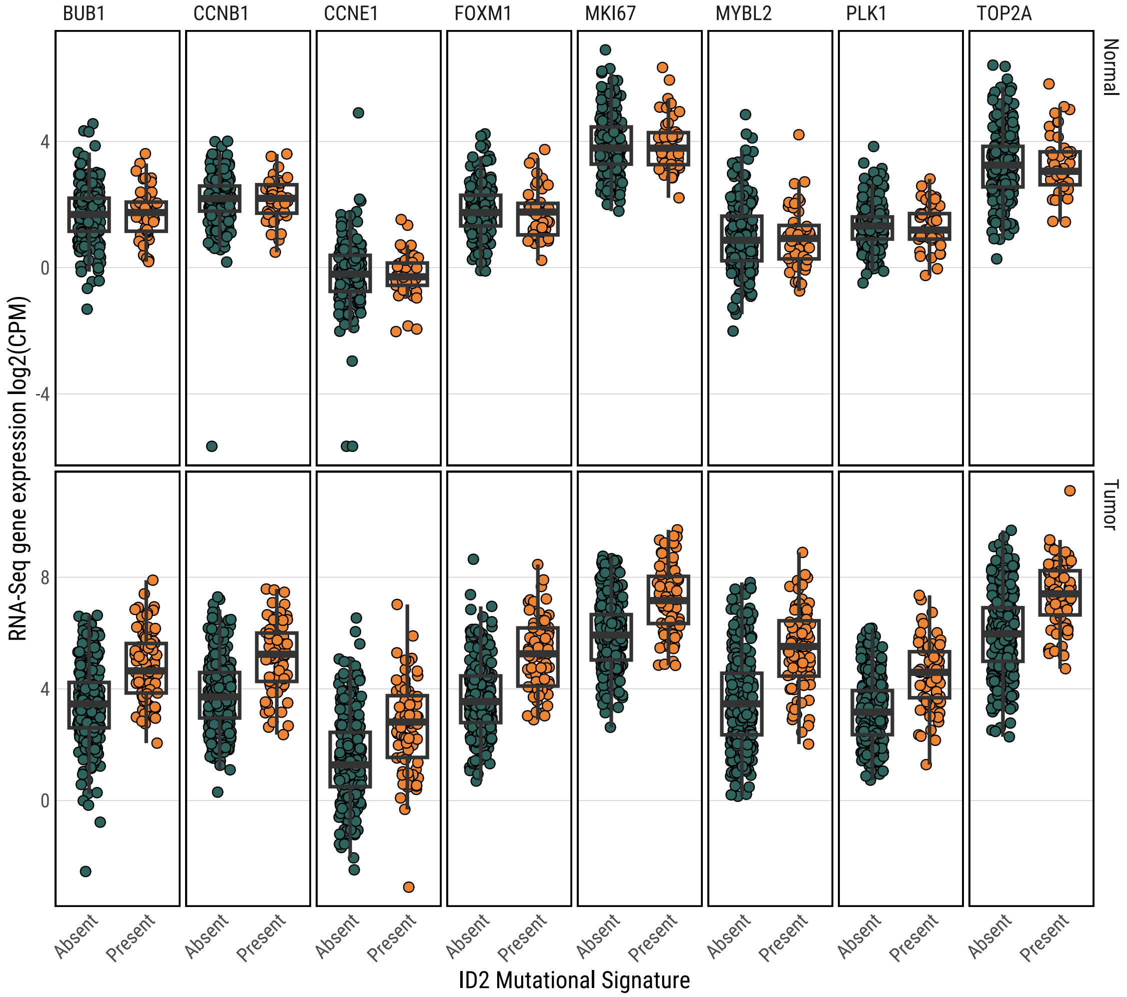

Supplementary Fig. 22

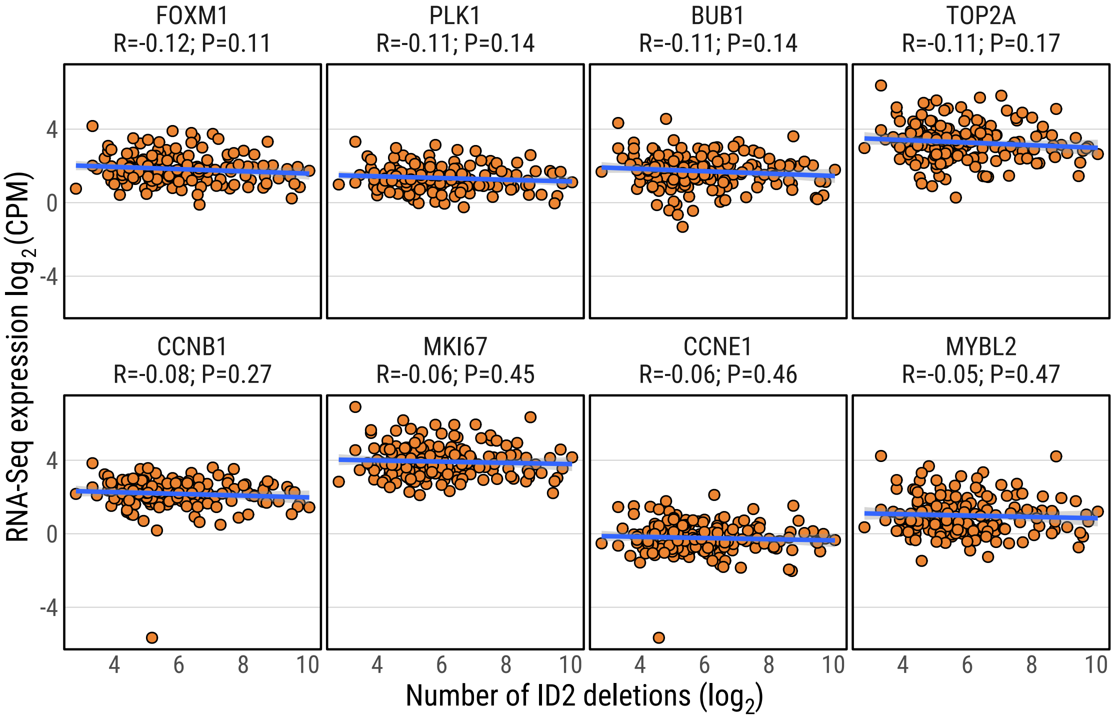

Supplementary Fig. 23

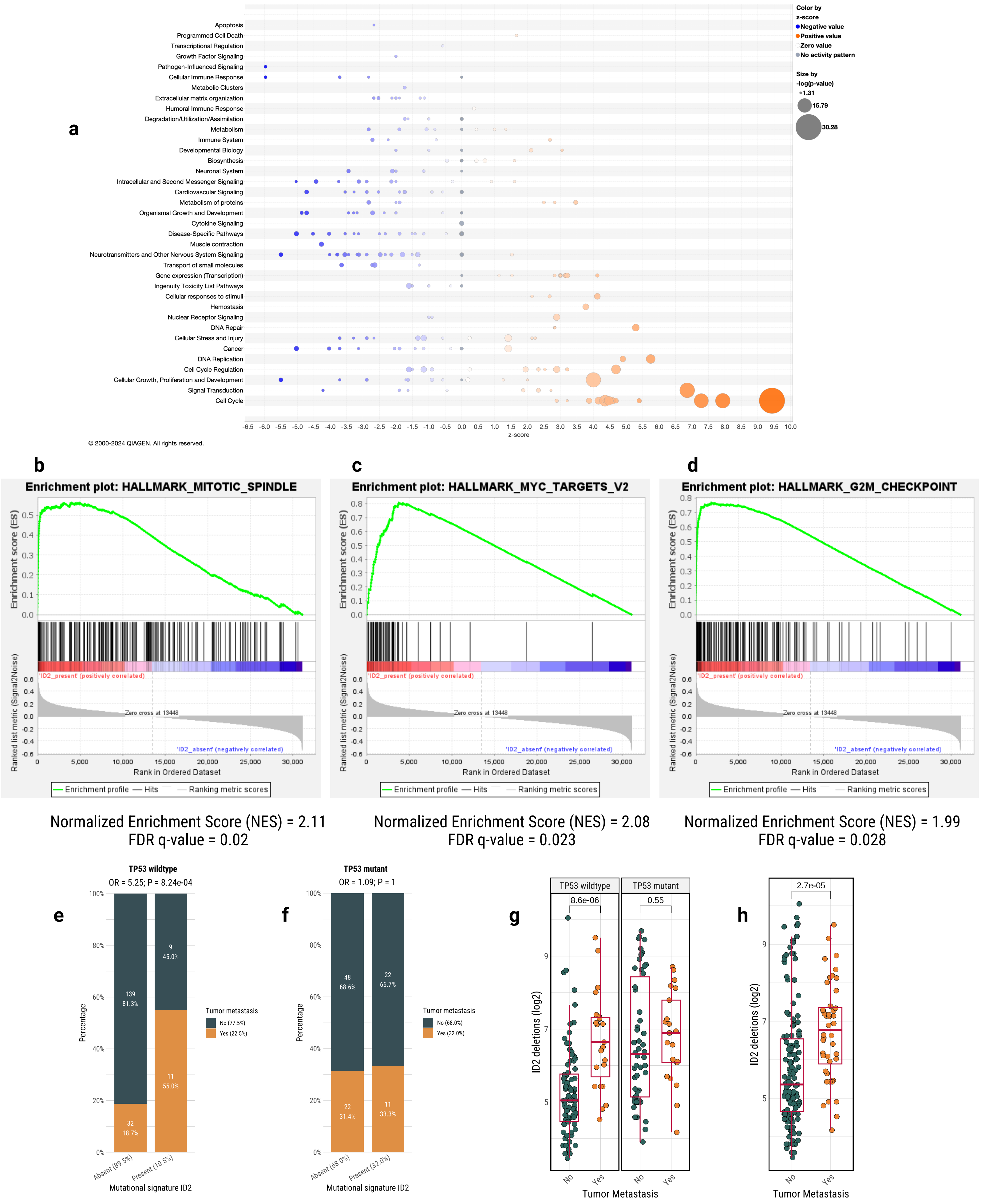

Supplementary Fig. 24

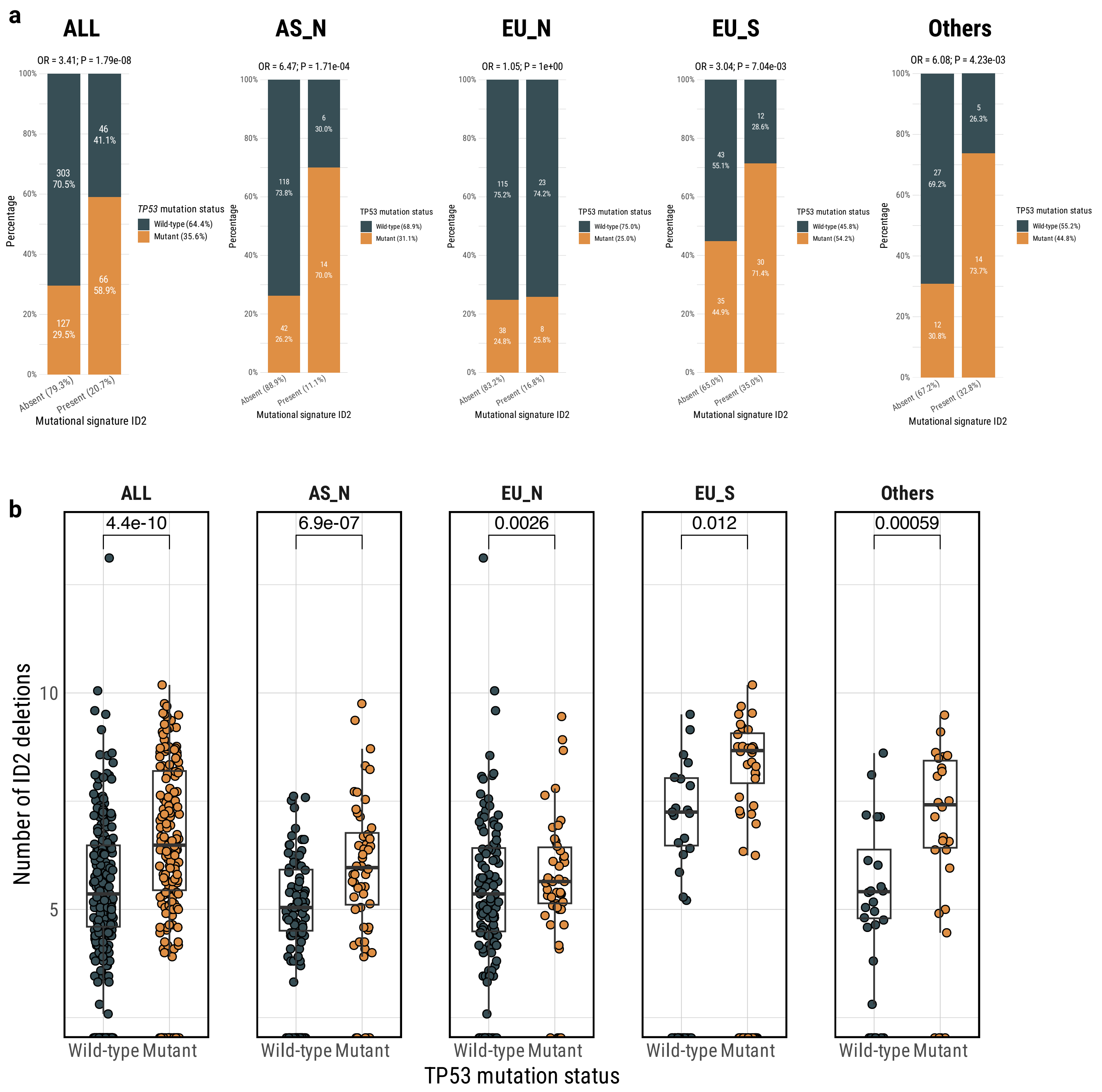

Supplementary Fig. 25

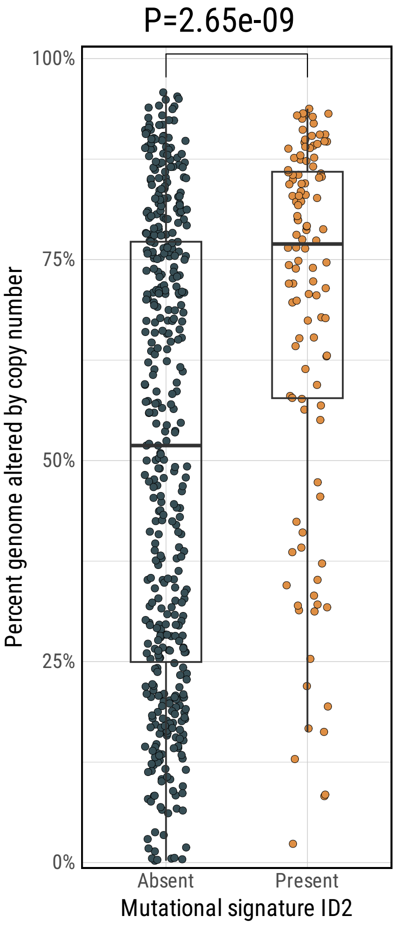

Supplementary Fig. 26

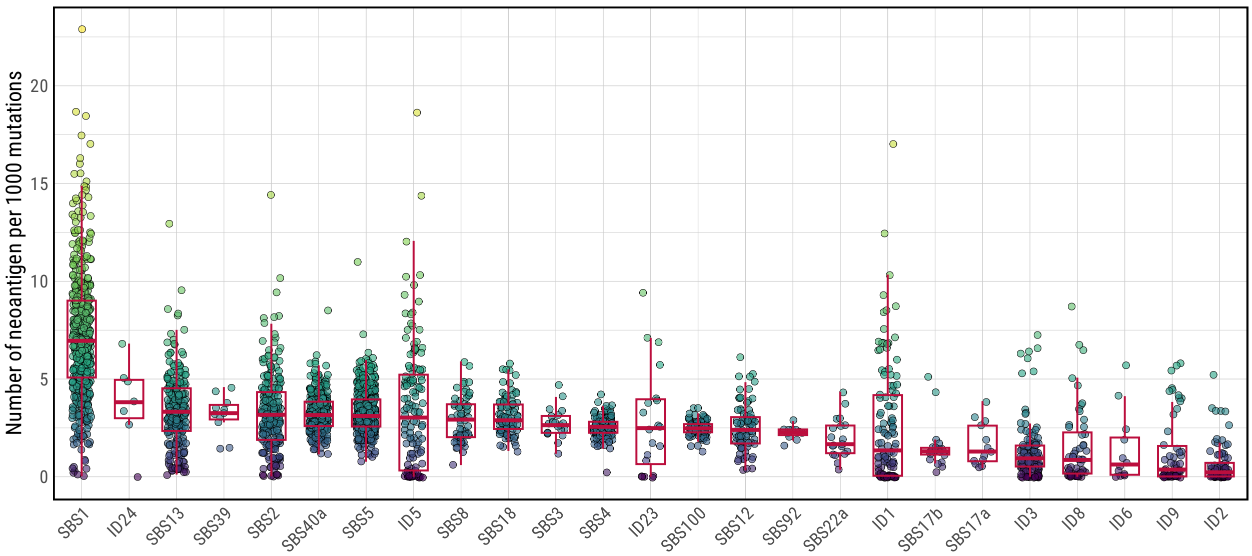

Supplementary Fig. 27

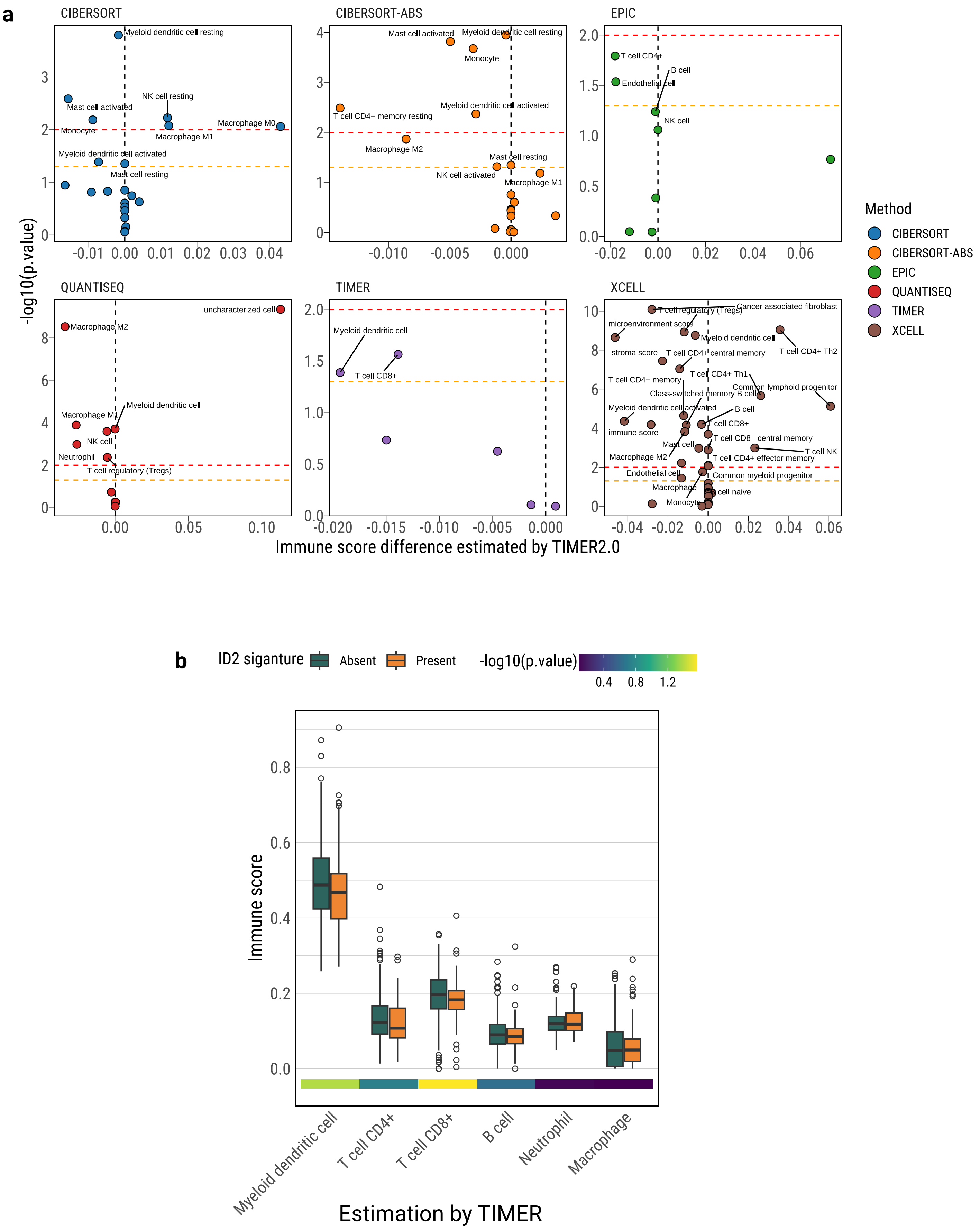

Supplementary Fig. 28

a

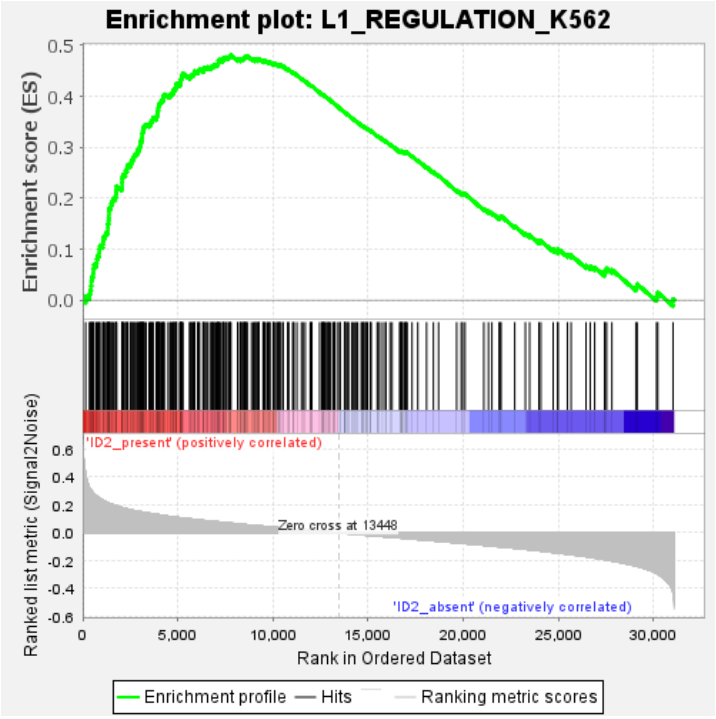

Normalized Enrichment Score (NES) = 2.02
FDR q-value = 0.025

b

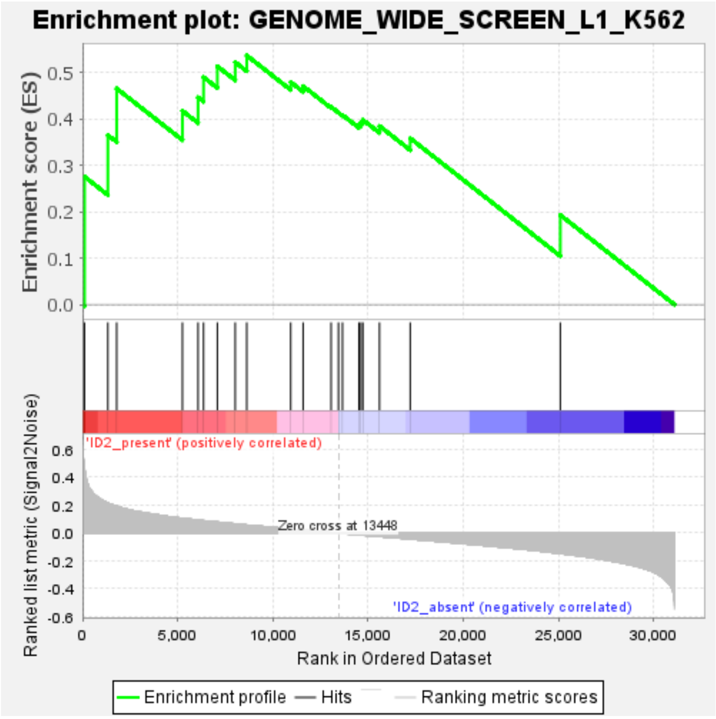

Normalized Enrichment Score (NES) = 1.80
FDR q-value = 0.042

Supplementary Fig. 29

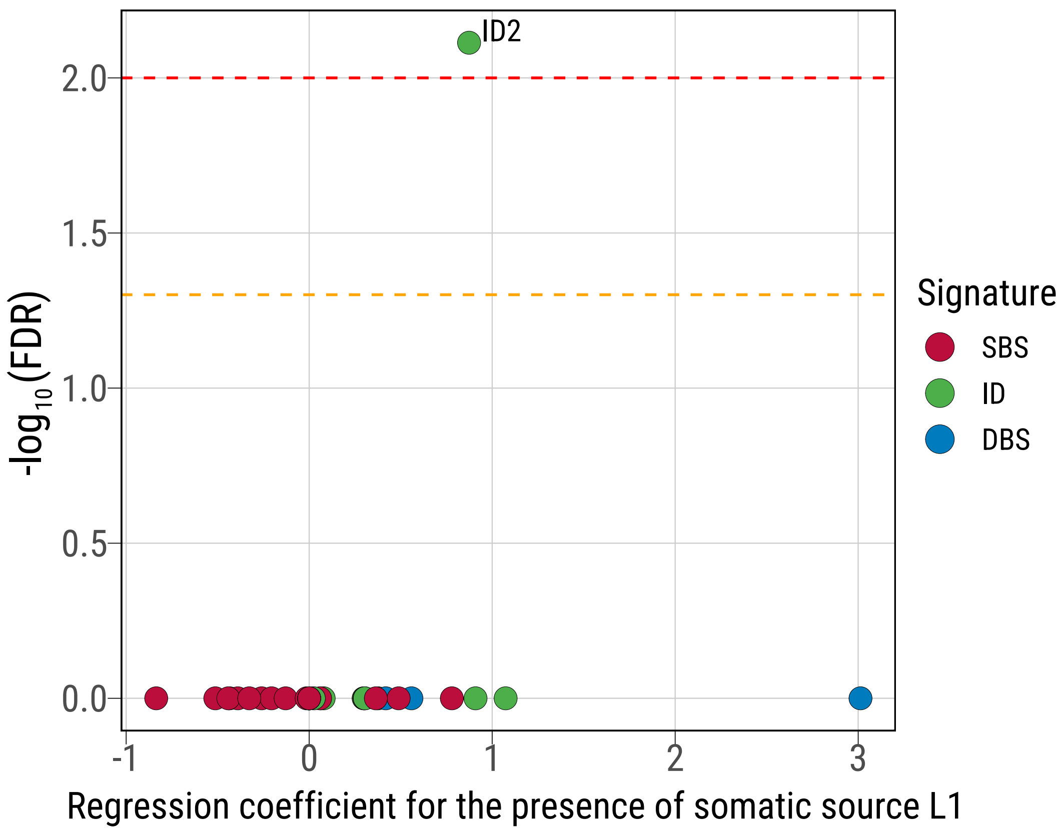

Supplementary Fig. 30

a

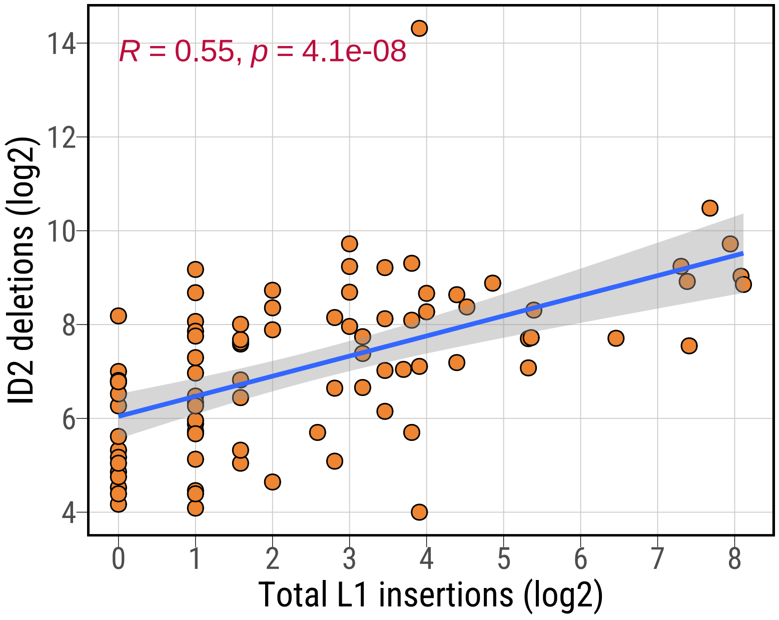

b

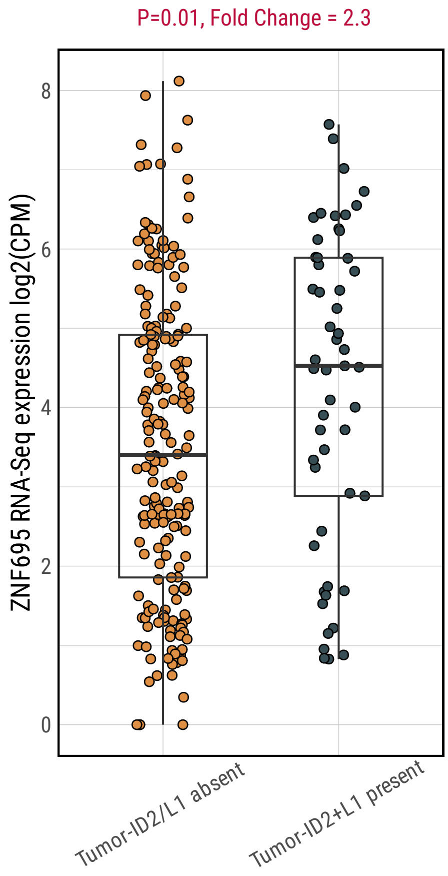

Supplementary Fig. 31

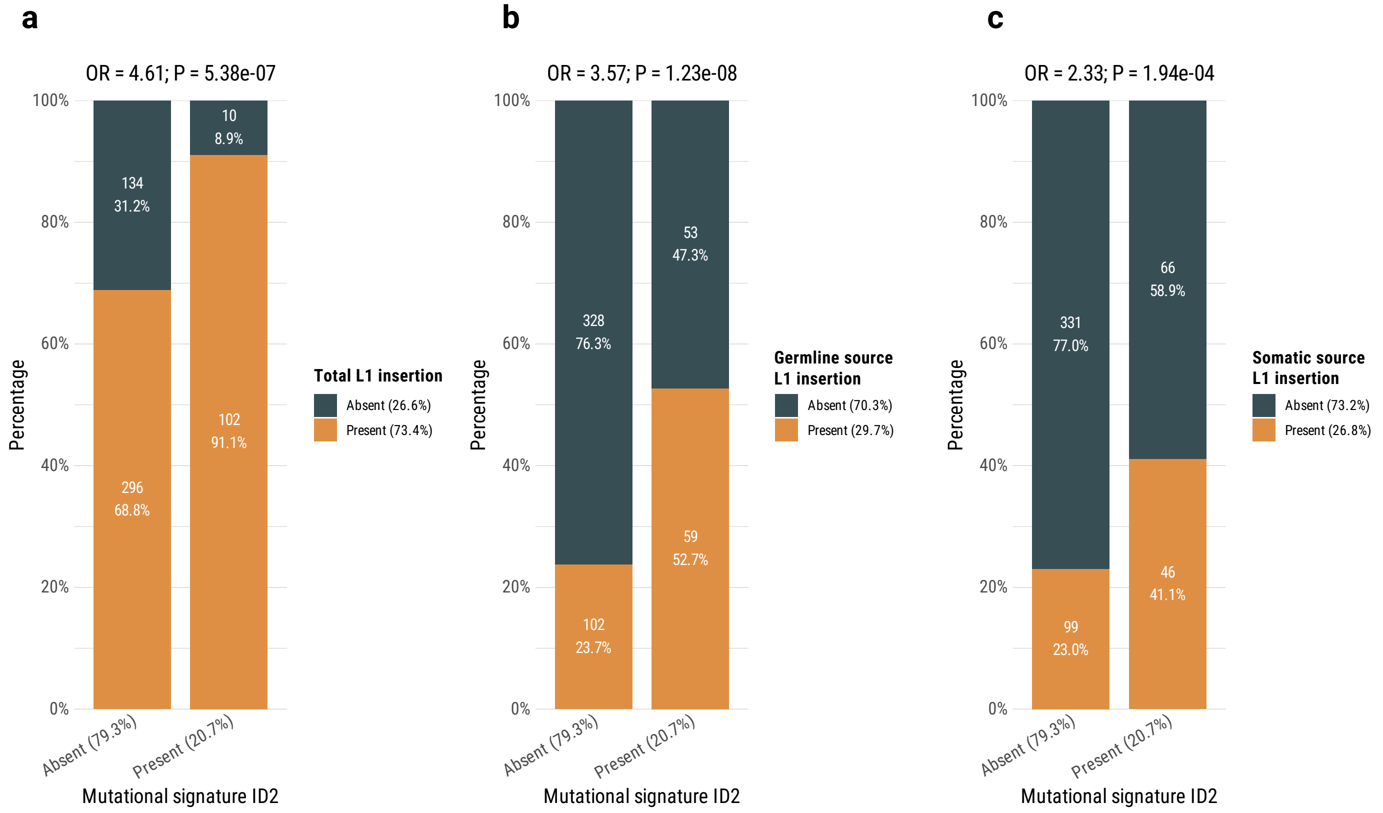

Supplementary Fig. 32

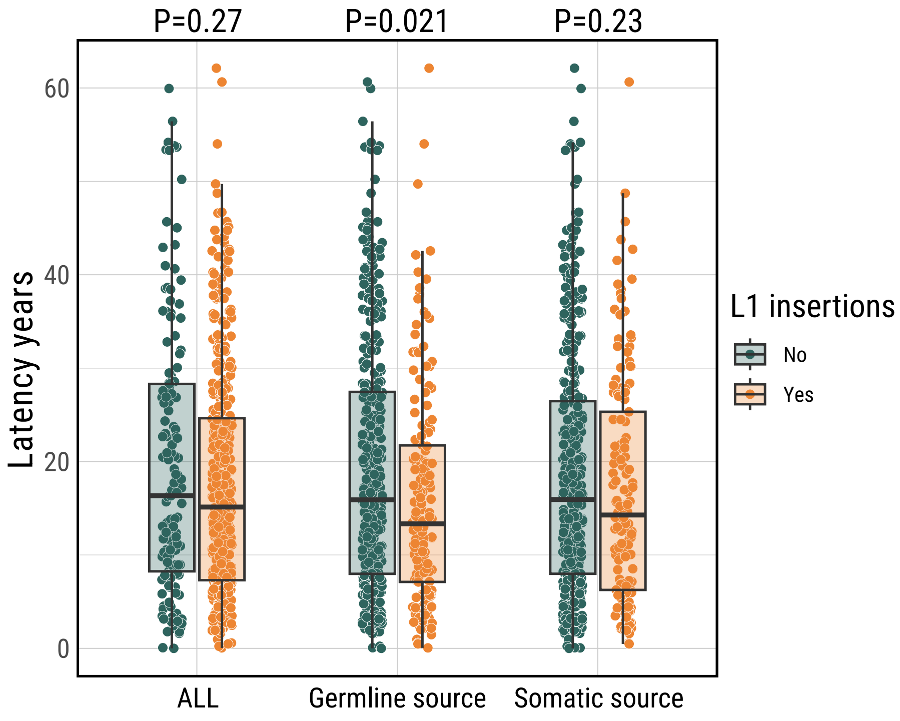

Supplementary Fig. 33

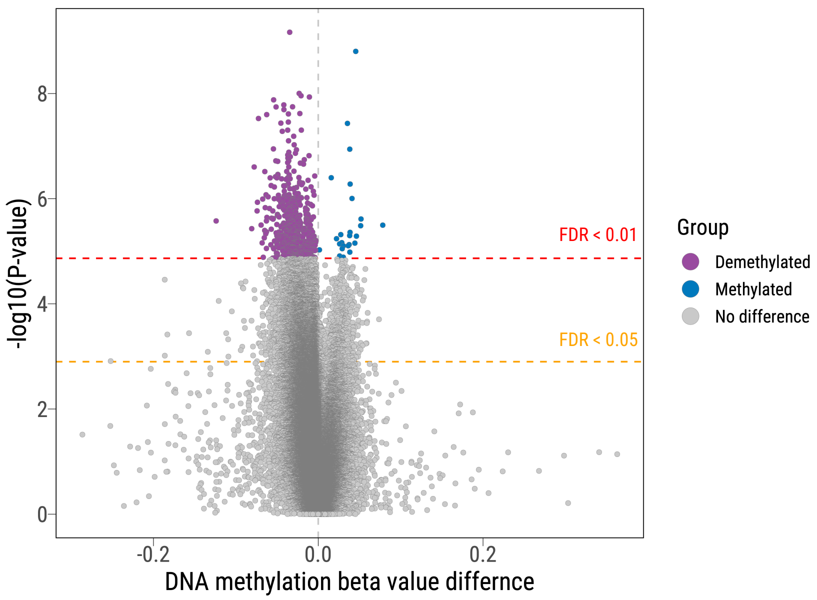

Supplementary Fig. 34

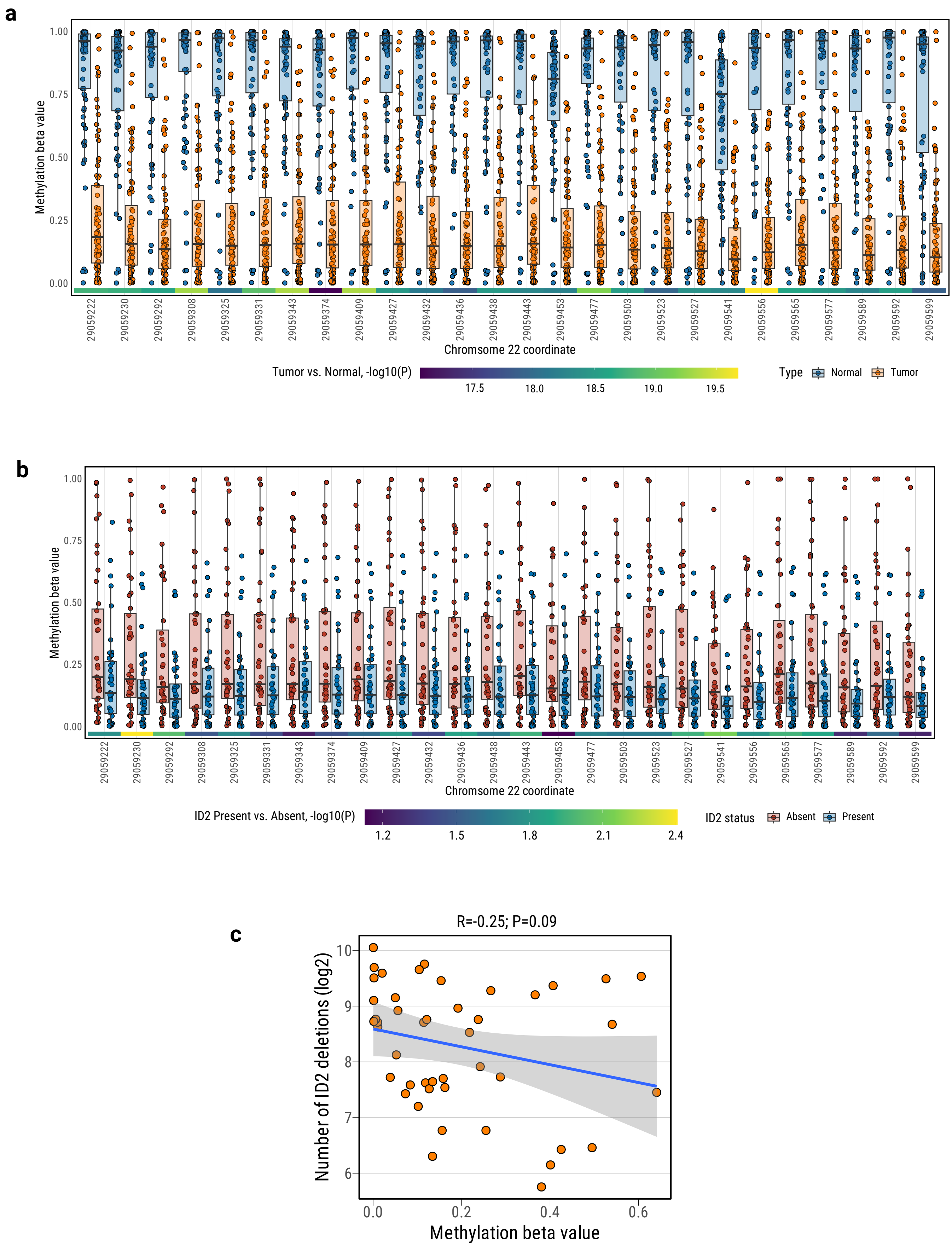

Supplementary Fig. 35

a

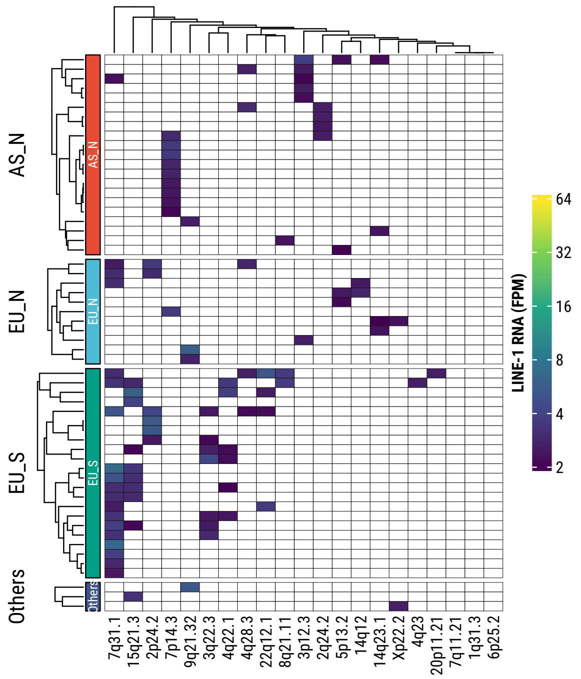

b

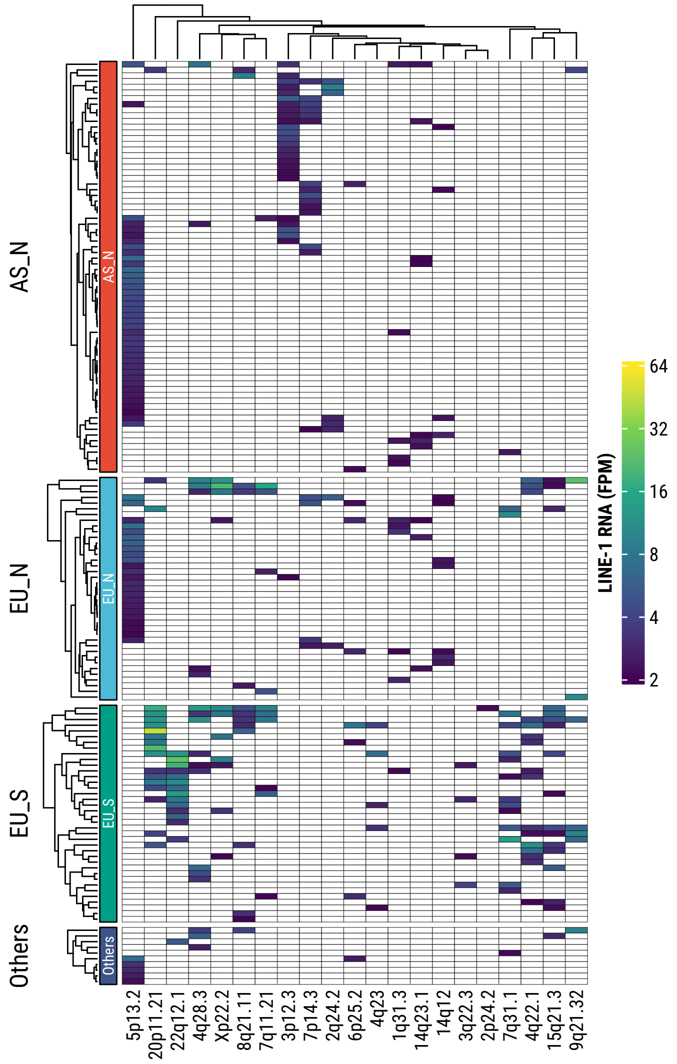

Supplementary Fig. 36

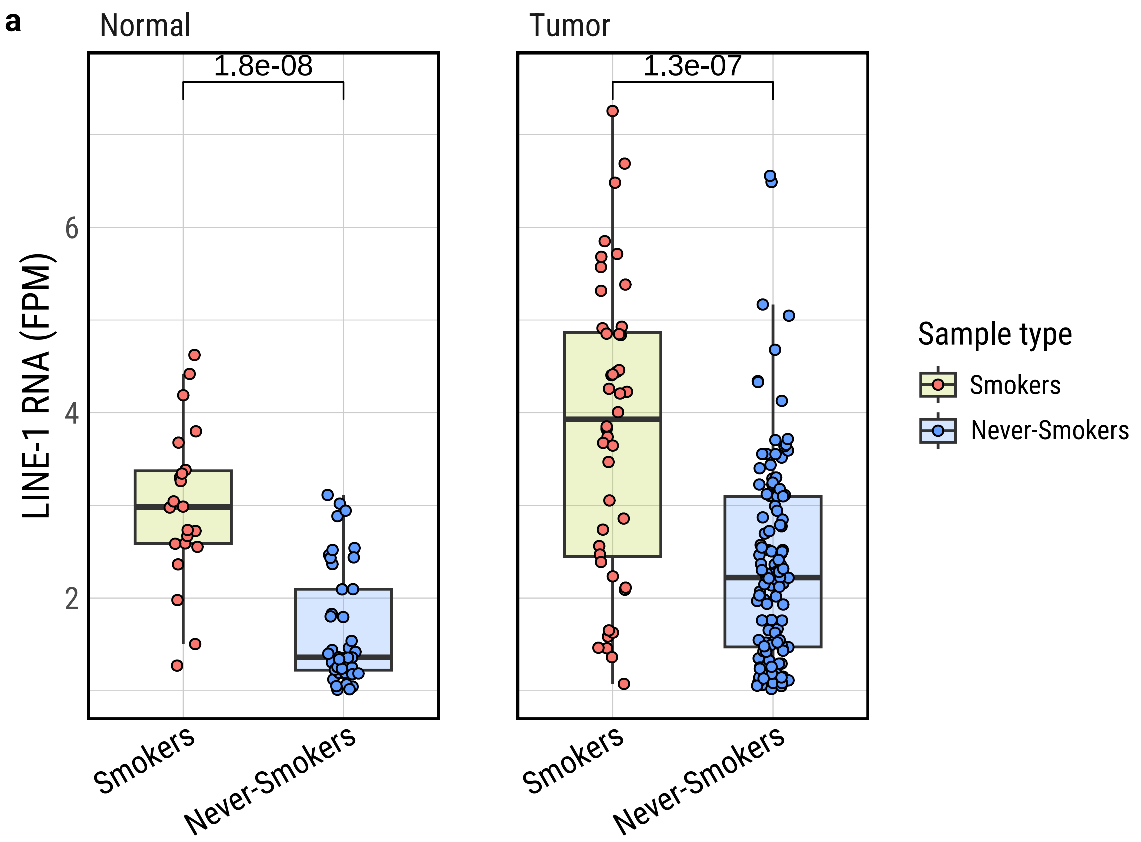

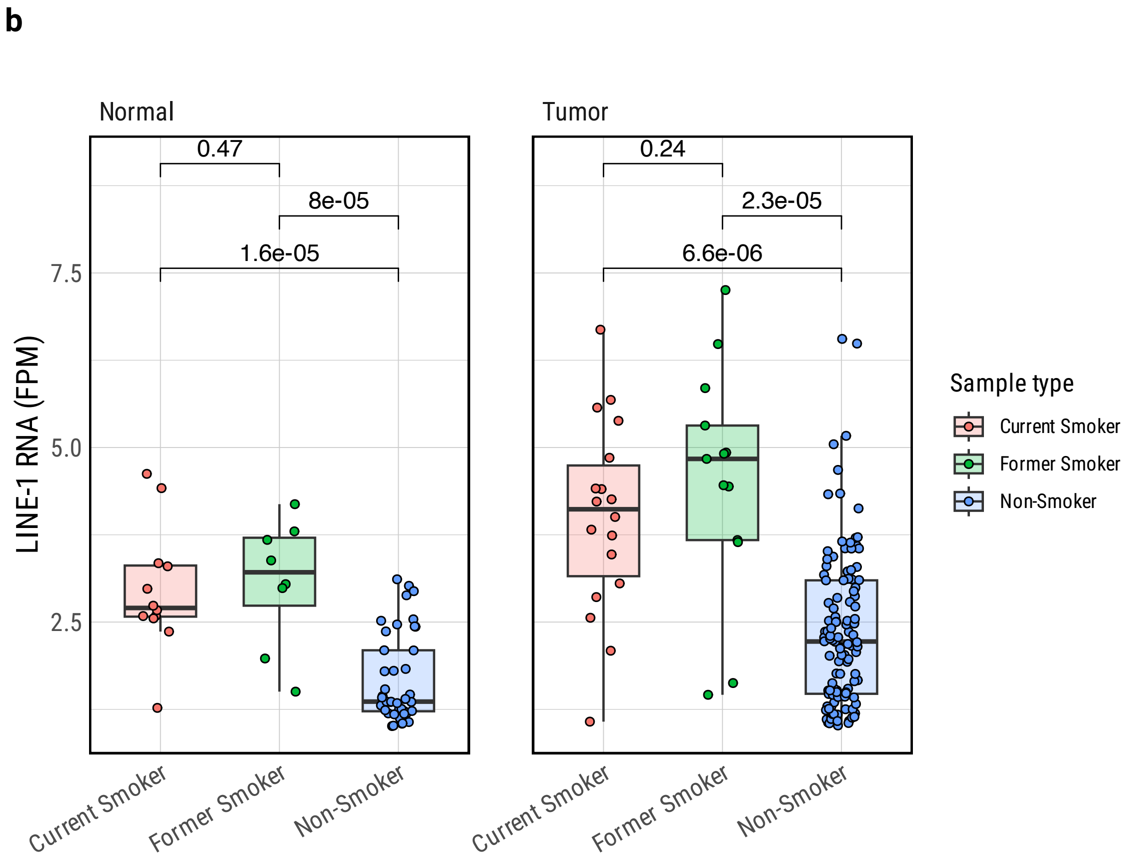

Supplementary Fig. 37

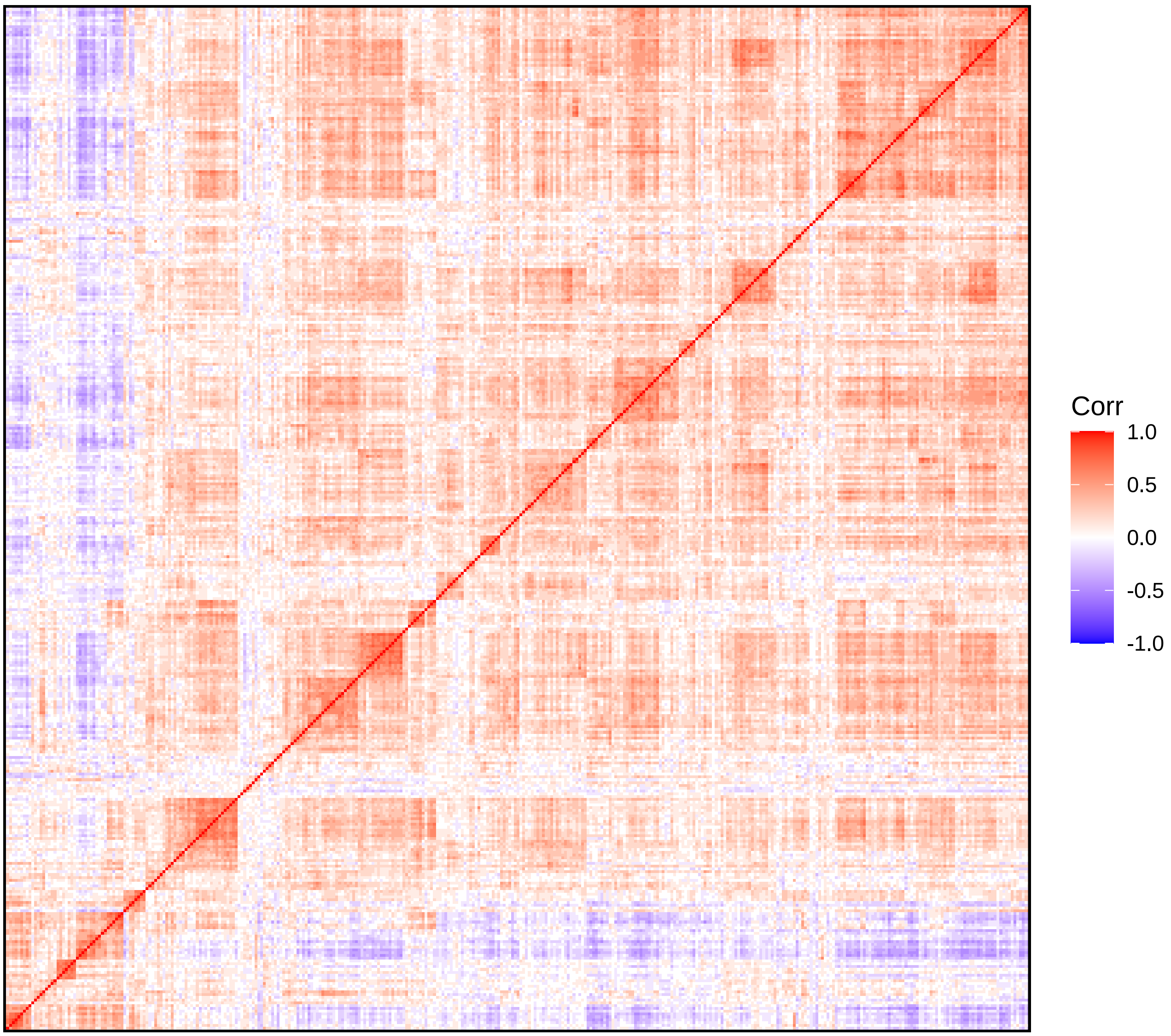

Supplementary Fig. 38

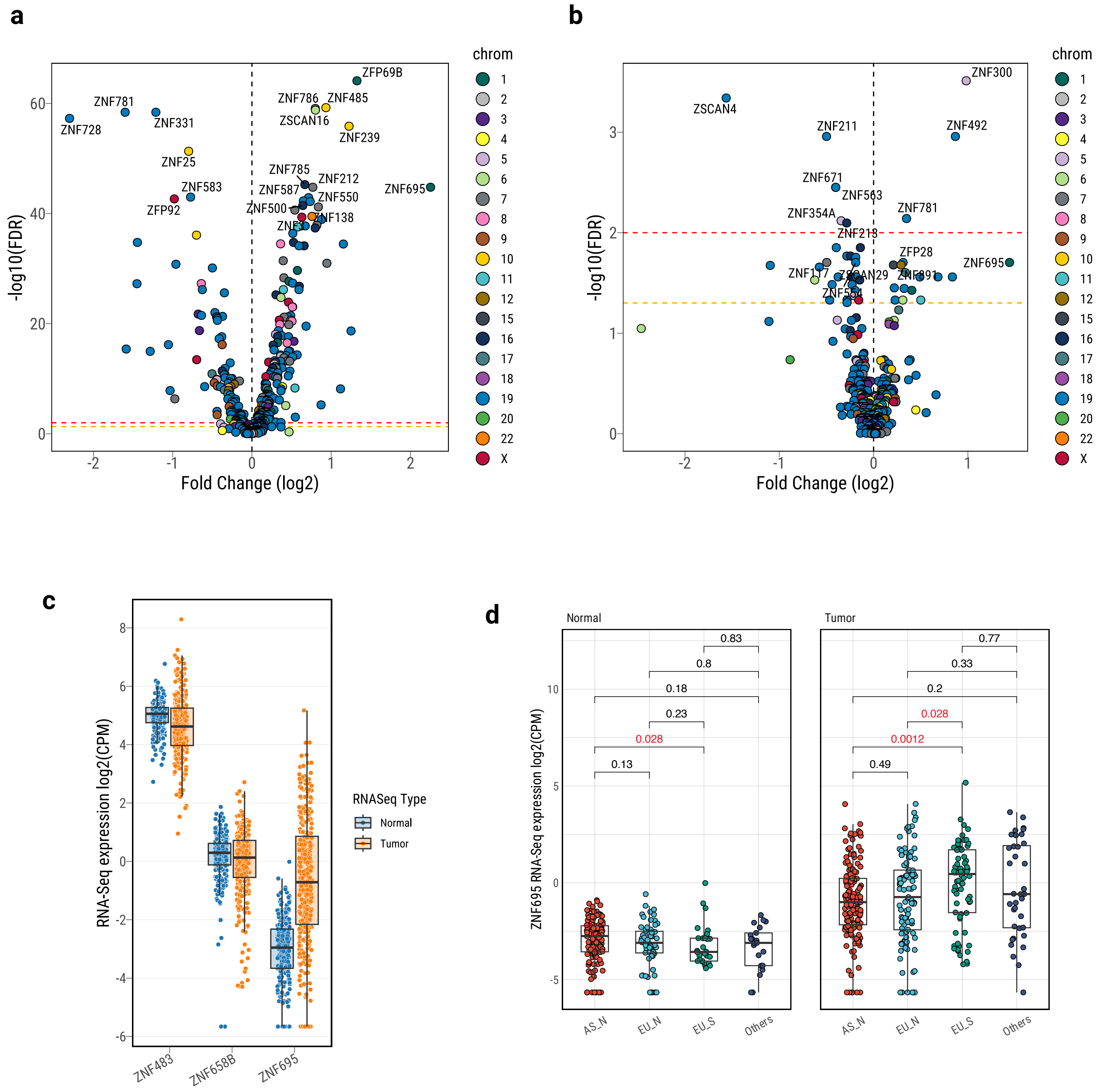

Supplementary Fig. 39

a

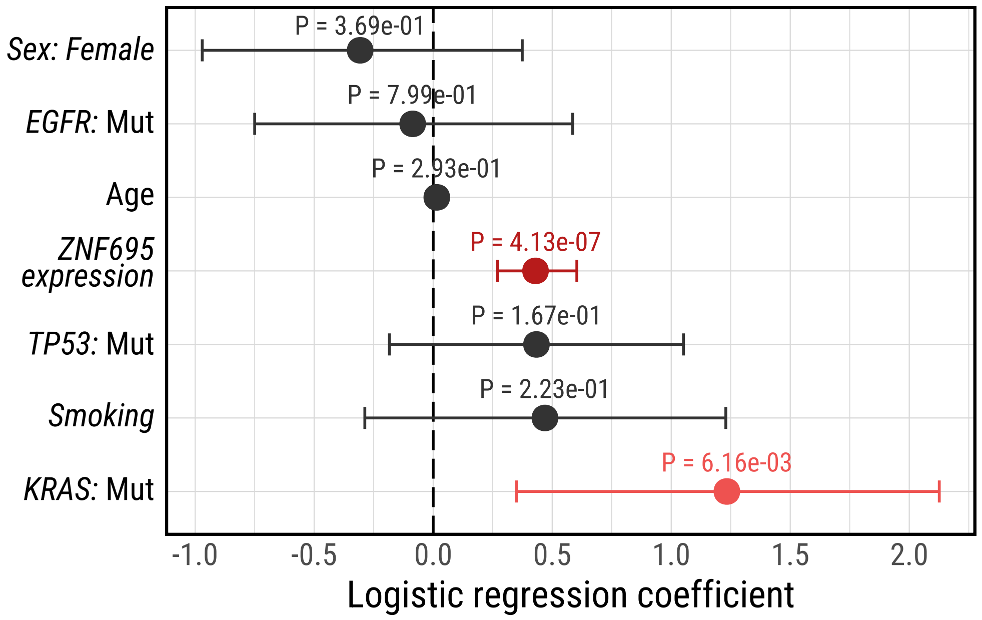

b

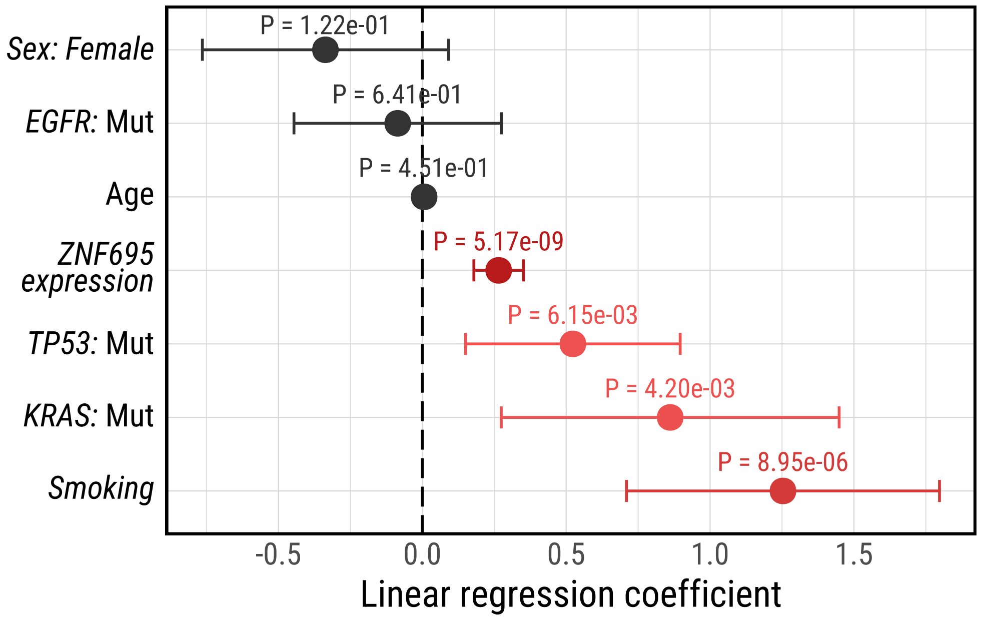

Supplementary Fig. 40

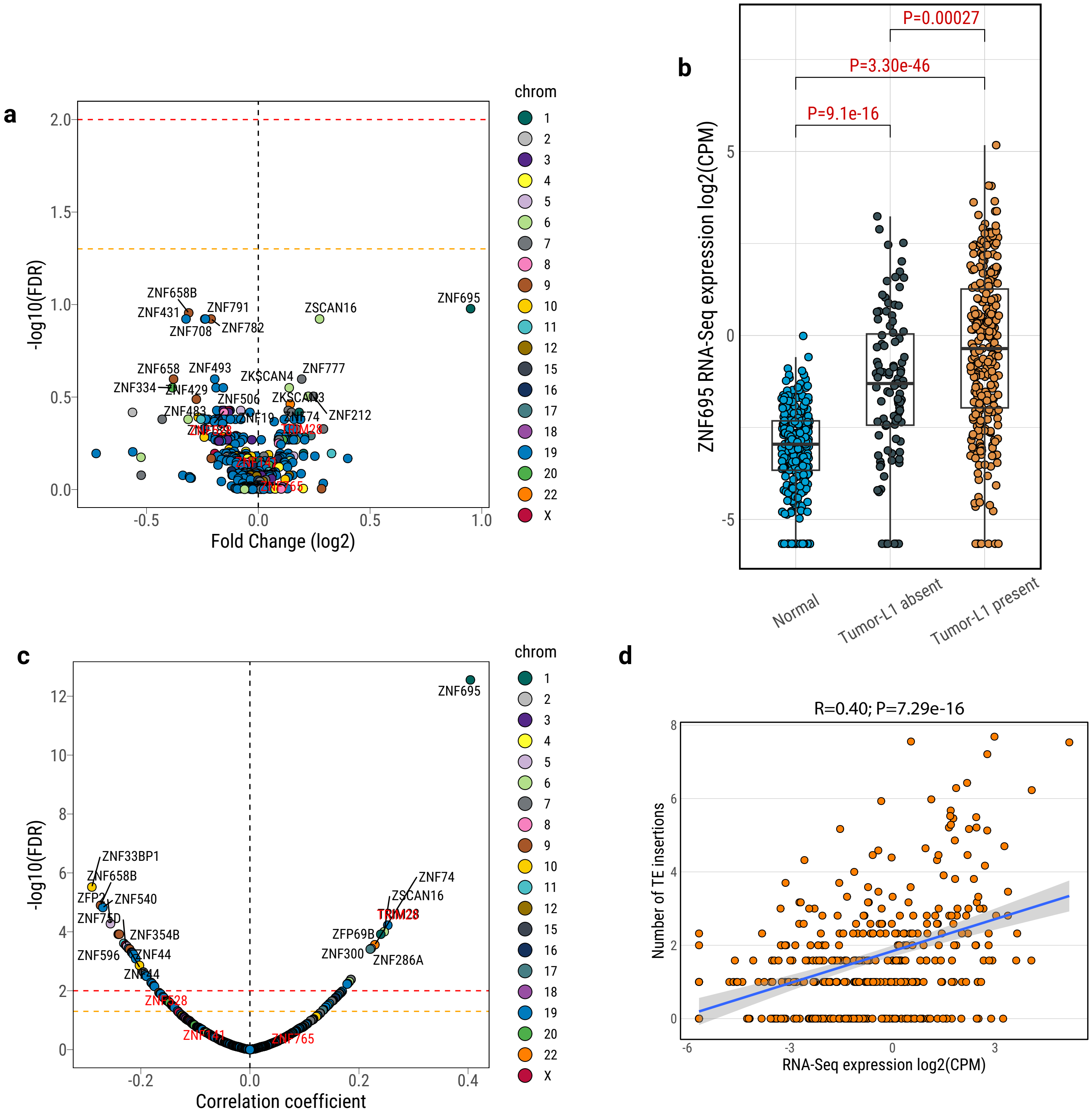

Supplementary Fig. 41

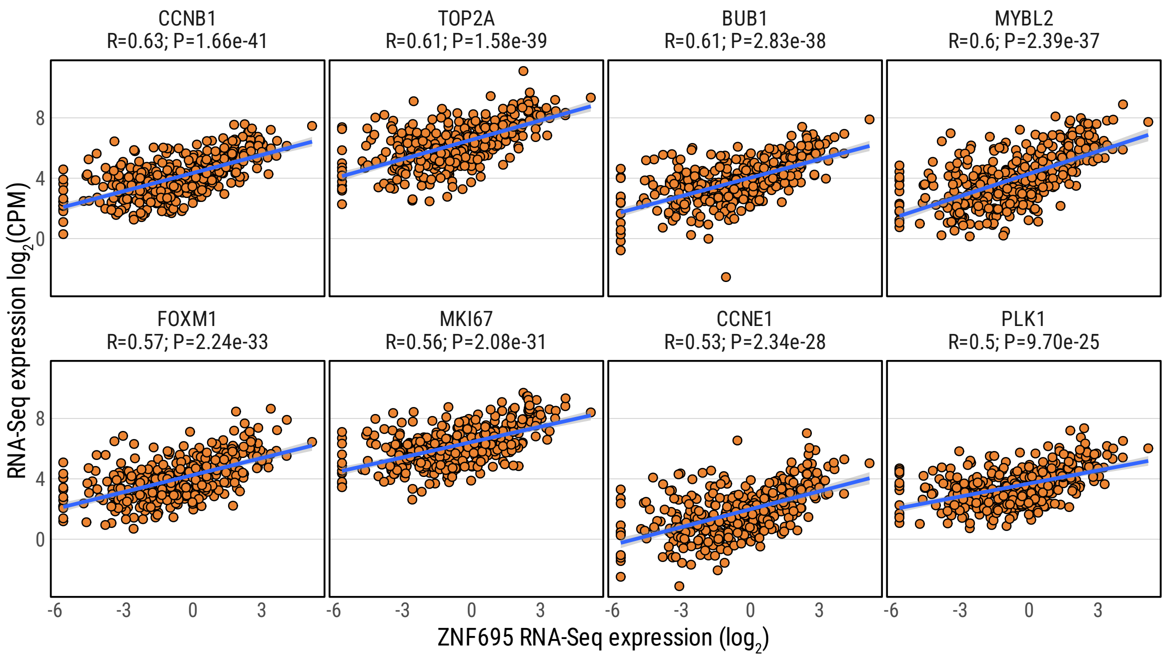

Supplementary Fig. 42

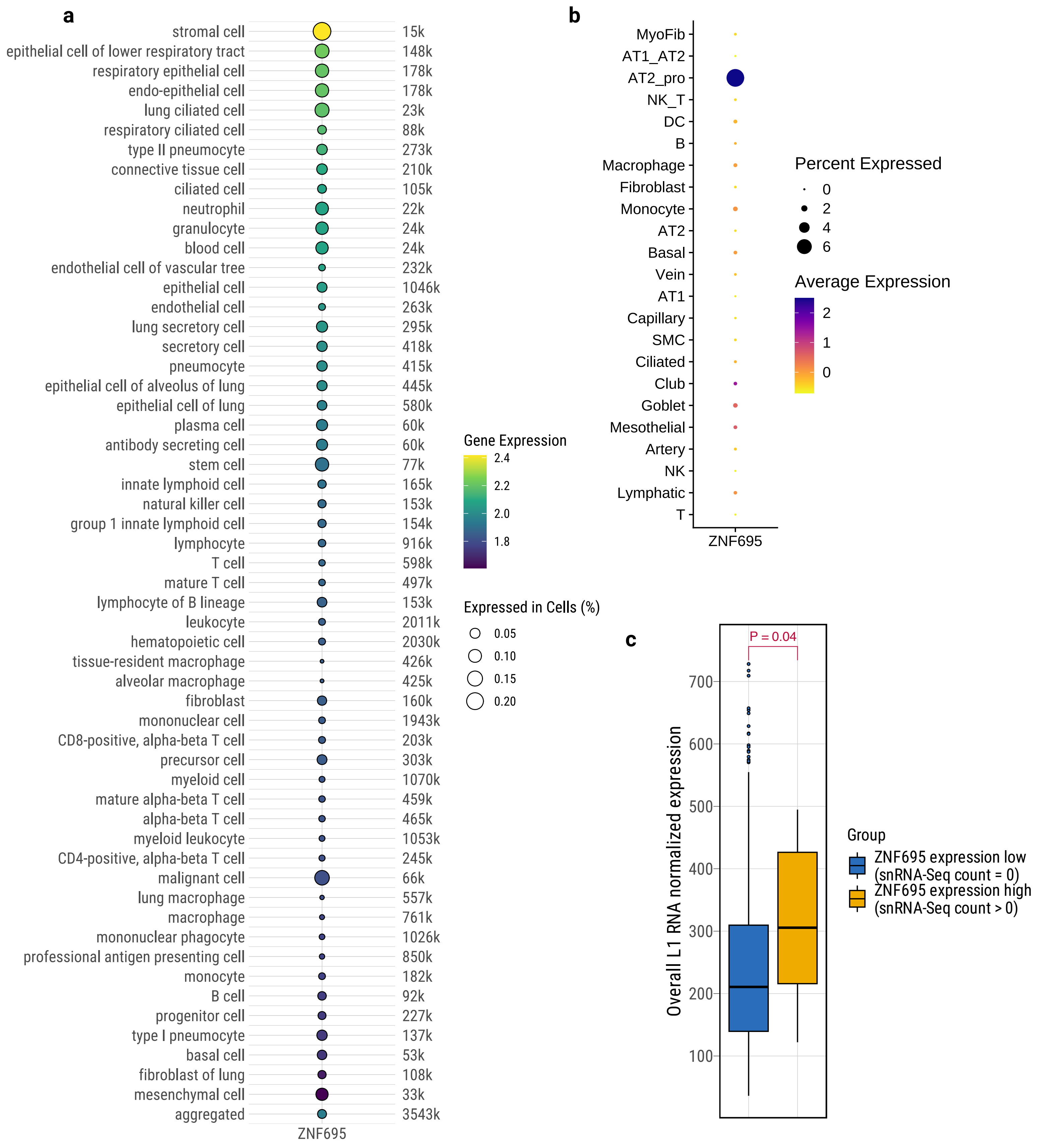

Supplementary Fig. 43

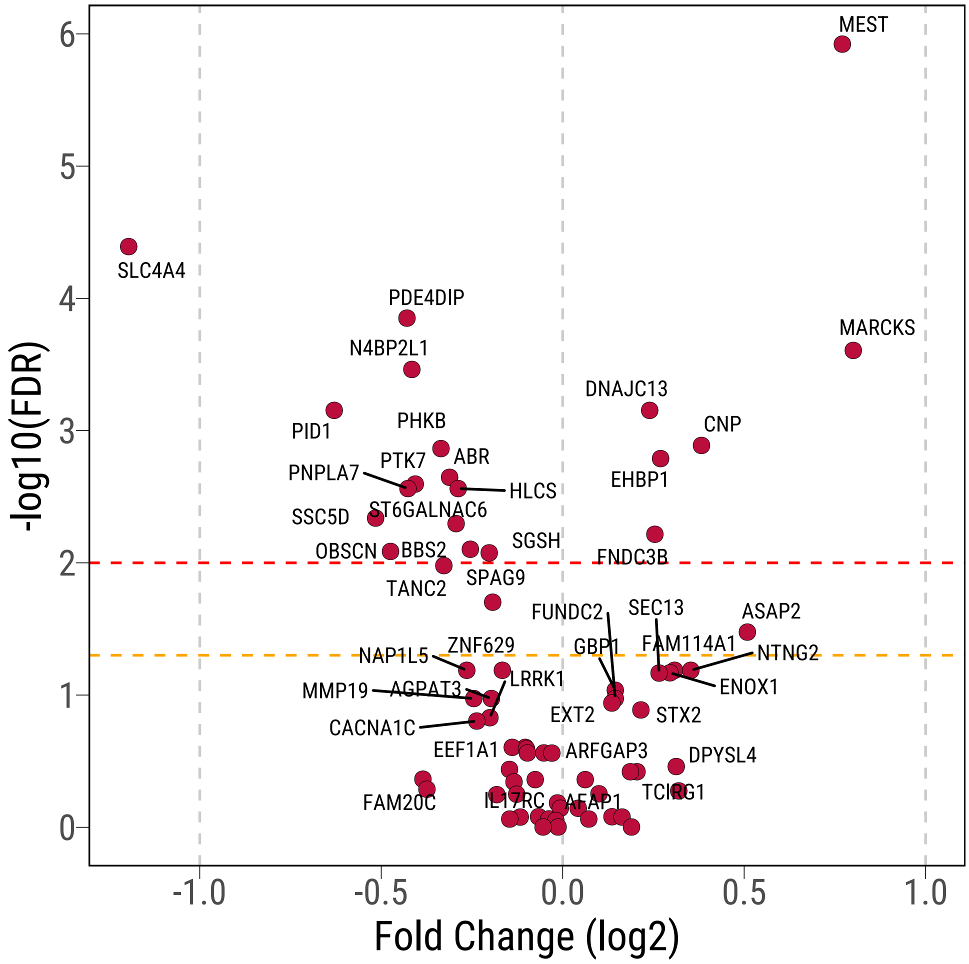

Supplementary Fig. 44

Only show junctions with depth >10% of max sequencing depth

a

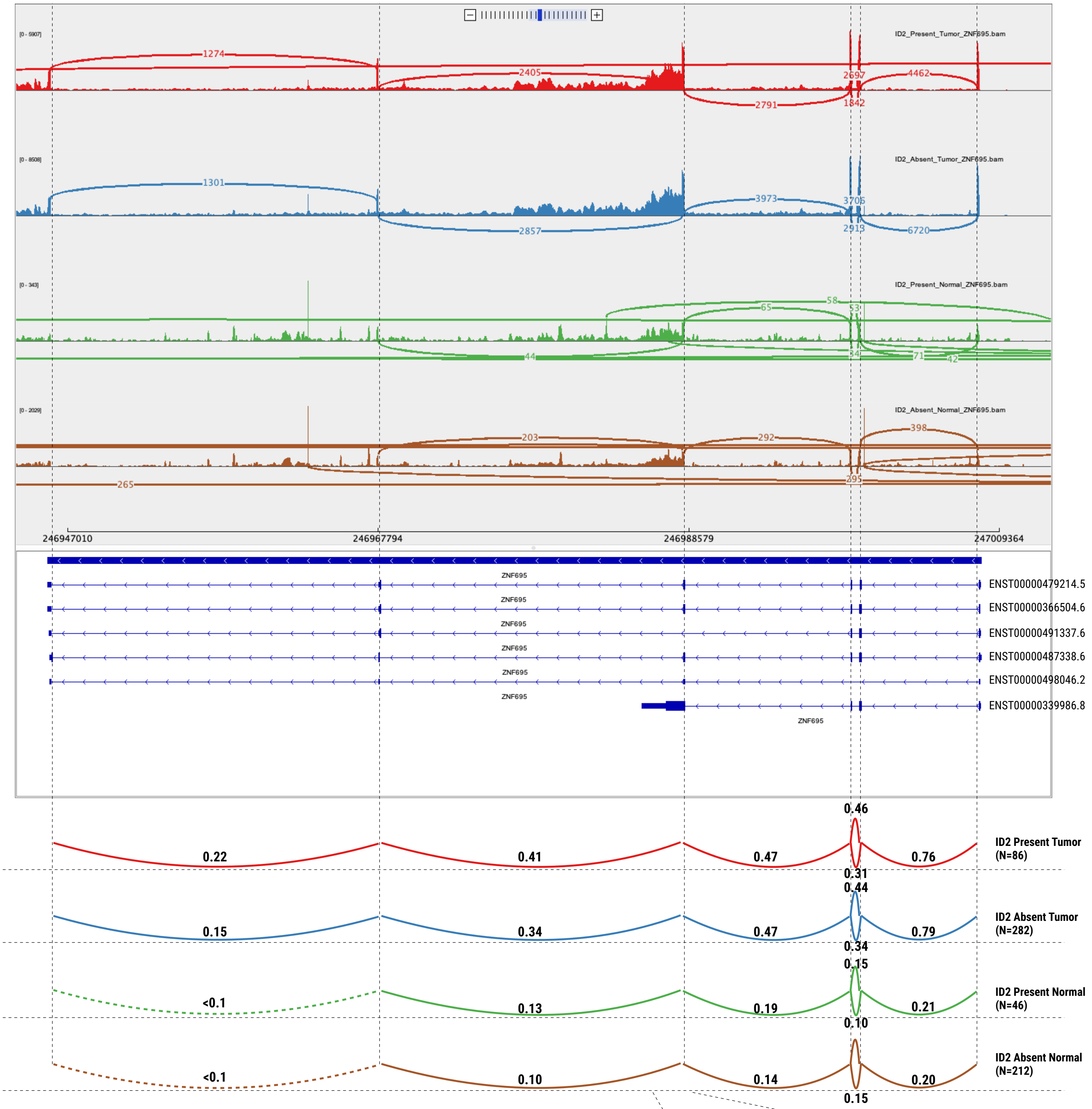

b

c

Transcript ID	Transcript Name	Refseq	Amino Acide	Protein Domain	Protein Type
ENST00000479214.5	ZNF695-203	NR_037894	121	KRAB	nonsense_mediated_decay
ENST00000366504.6	ZNF695-202	-	133	KRAB	nonsense_mediated_decay
ENST00000491337.6	ZNF695-205	-	88	KRAB	nonsense_mediated_decay
ENST00000487338.6	ZNF695-204	NM_001204221	172	KRAB	protein_coding
ENST00000498046.2	ZNF695-206		-	-	processed_transcript
**ENST00000339986.8**	**ZNF695-201**	NM_020394	**515**	KRAB and Zinc-finger	**protein_coding**

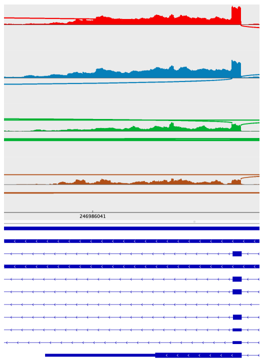

Supplementary Fig. 45

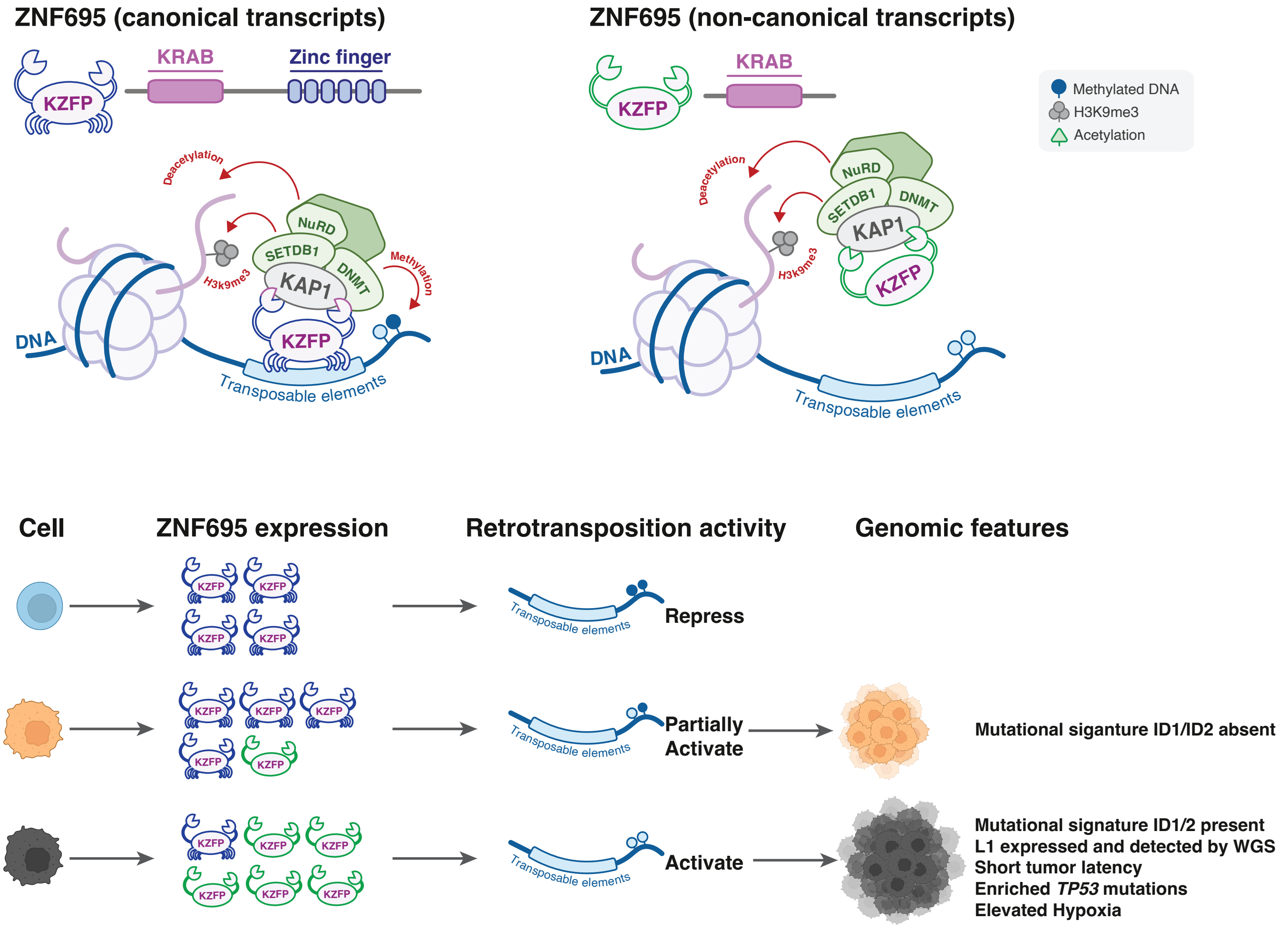

Supplement: Supplement 1 — Supplementary Fig. 1: Power analysis for detecting a diverse clonal architecture. a) The scatter plot illustrates the relationship between the number of reads per chromosome copy (NRPCC) and the total detected single nucleotide variants (SNVs). Our ability to detect subclones relies not on the number of identified SNVs, but on the number of reads per tumor chromosomal copy. NRPCC accounts for tumor purity, ploidy, and sequencing coverage. b) The minimum cancer cell fraction (CCF) of the detected clusters in each tumor is plotted against NRPCC. To mitigate biases, we exclusively considered tumors with NRPCC ≥ 10. In these tumors, our analysis is sufficiently powered to identify a subclone with a CCF ≥ 30%. The suggested NRPCC threshold is denoted by the dashed line. Supplementary Fig. 2: Detailed sample and data information for LUAD tumor evolution analysis. a) Summary of multi-omics data stratified by group information. b) proportion of samples within each group. c) Ancestry inference based on comparisons with samples from the 1000 Genome Project. Supplementary Fig. 3: Detection of whole genome doubling (WGD) events. a) Tumor samples with and without WGD (nWGD) are distinguished based on their ploidy and the fraction of the genome exhibiting loss of heterozygosity (LOH). The initial demarcation line between WGD and nWGD tumors, established by the PCAWG study26, is represented as y = 2.9 − 2x. b) The fraction of the autosomal genome with a major copy number (MCN) of two or greater displays a bimodal distribution, highlighting the distinct separation between WGD and nWGD tumors. Supplementary Fig. 4: Overview of the molecular timing distribution of copy number gains per chromosome. a) Pie charts depict the distribution of the inferred mutation time for a given copy number gain in a group. Green denotes early clonal gains, with a gradient to purple for late gains. The size of each pie chart is proportional to the frequency of recurrence of each event. b) Comparison of [file media-1.pdf]
